# AgroPatch AutoStrike for adversarial patch attacks on plant disease detection models

**DOI:** 10.1016/j.isci.2026.117054

**Published:** 2026-08-04

**Authors:** Yuting Wu, Mingxing Wang, Xiu Jin, Yue Dai, Liang Liu, Wenjie Yuan, Yong Ye, Zhengkang Zhu, Lichuan Gu, Youtao Zhang

**Affiliations:** 1 School of Information and Artificial Intelligence, Anhui Agricultural University, Hefei, Anhui 230036, China; 2 Department of Computer Science, Illinois Institution of Technology, Chicago, IL 60616, USA; 3 Department of ECE, University of Pittsburgh, Pittsburgh, PA 15260, USA; 4 Department of CS, University of Pittsburgh, Pittsburgh, PA 15260, USA

**Keywords:** agricultural disease identification, vision transformers, adversarial attack, patch attack, loss function

## Abstract

Plant diseases threaten food security and cause substantial economic losses, motivating artificial intelligence for automated detection. Although vision transformers (ViTs) have shown promising performance in agriculture, their vulnerability to adversarial patch attacks remains underexplored. Existing patch attacks developed for generic images are less effective for plant disease images. We propose AgroPatch-AutoStrike (APA), an adversarial patch attack framework for ViT-based plant disease detection. APA combines the variance driven feature patch attack (VDFPA) with the adversarial focal loss (AFL) to improve patch placement and emphasize hard to attack categories. On the cassava leaf disease dataset, APA reduces the robust accuracy (RA) of most ViT models to nearly zero using four patches covering 2% of the image. Compared with patch-fool and LaVAN, APA achieves 1–2% lower RA with comparable inference time. These findings reveal robustness gaps in plant disease detection models and support domain specific adversarial robustness evaluation for agricultural AI.

## Highlights

• APA evaluates patch attacks on plant disease detection models
• VDFPA improves patch placement using variance and texture cues
• Four APA patches reduce ViT robust accuracy to nearly zero
• APA supports robustness evaluation for agricultural AI models

## Graphical abstract

**Figure:**
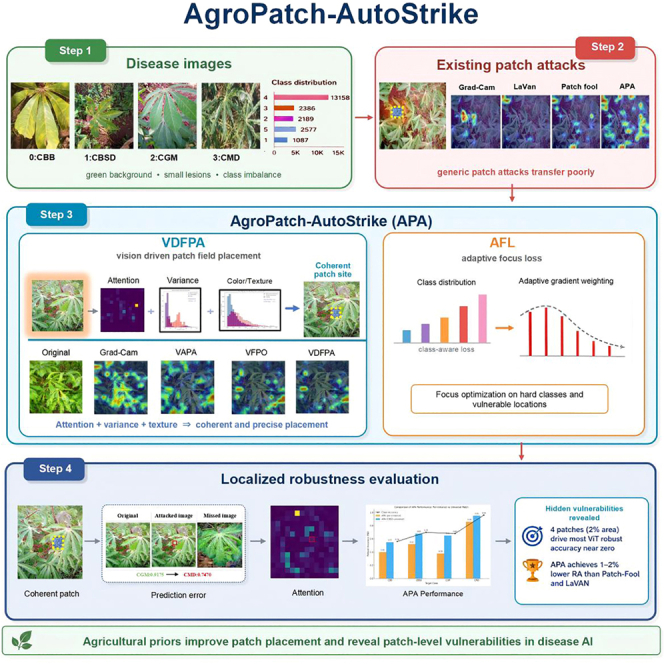

## Teaser

Plant pathology; Agricultural science; Agronomy; Agricultural tool

## Introduction

Agriculture plays a pivotal role in the global economy, providing essential food supplies and various raw materials. While the World Bank reported that the global daily food production reached 8.4 million metric tons in 2022, food production is vulnerable to pests and diseases, resulting in substantial economic losses such as reduced food production and destruction of farmland.^1^^,^^2^ With agricultural environments becoming increasingly complex, accurate and timely identification of plant diseases is critical for ensuring sustainable food production.^3^ In recent years, artificial intelligence (AI) techniques, particularly deep learning-based AI models, have shown great promise in automating plant disease diagnosis from images, offering improved efficiency and accuracy in agricultural monitoring tasks.^4^^,^^5^^,^^6^^,^^7^ Recent studies have also explored plant disease detection models for edge computing devices, indicating that disease recognition systems are moving toward resource-constrained and deployable agricultural scenarios.^8^

Most existing studies primarily focus on improving classification accuracy and defending against general security vulnerabilities posed by adversarial samples. These samples are carefully crafted perturbations that are imperceptible to the human eye but can significantly mislead model predictions. Unfortunately, limited attention is paid to the security vulnerabilities in agricultural scenarios. In real-world agricultural applications, such attacks can result in incorrect disease diagnoses, misallocation of resources, and reduced crop yields.^9^^,^^10^ Despite these risks, the adversarial robustness of plant disease detection models under realistic and physically plausible conditions remains largely understudied.

Agricultural disease detection has evolved from manual inspection to computer vision (CV)-based solutions. Early techniques employed classifiers such as support vector machines (SVMs) and artificial neural networks (ANNs).^11^^,^^12^ And clustering-based methods, such as K-means clustering, are used for rice leaf disease identification.^13^ More recently, convolutional neural networks (CNNs) have significantly advanced performance in plant disease recognition.^14^^,^^15^^,^^16^ Techniques such as GhostNet^17^ and transfer learning frameworks^18^ further enhance accuracy and computational efficiency. Nonetheless, CNNs often struggle with visual noise, occlusion, and data distribution shifts prevalent in agricultural imagery.^5^^,^^19^

In contrast, ViTs utilize self-attention mechanisms to model long-range dependencies and global context,^20^ yielding superior performance in complex scenarios.^21^^,^^22^^,^^23^ For instance, SLViT^24^ outperforms CNNs on multiple benchmarks, offering both accuracy and efficiency benefits. The robustness of ViTs under adversarial attacks has also been studied.^25^^,^^26^^,^^27^^,^^28^ Although ViTs demonstrate resilience to natural noise, they are particularly vulnerable to localized adversarial patch attacks due to their reliance on token-level attention,^25^ e.g., perturbing even 1–3% of an image can severely degrade prediction accuracy.^26^^,^^29^ However, our preliminary studies using agricultural images reveal that traditional adversarial patch methods, such as patch-fool and LaVAN, while being highly effective on images from standard vision benchmarks, perform poorly when applied to agricultural disease images. For example, applying these attacks to cassava leaf datasets cannot lower the classification accuracy below 50%. Preliminary analyses reveal that the complex textures, irregular shapes, and heterogeneous backgrounds in agricultural images present natural defenses, reducing the effectiveness of generic patch attacks. That is, existing patch attack strategies do not transfer well to the agricultural domain. Thus, it is important to study domain-specific ViT-based attack methods that account for the visual patterns in plant disease datasets.

**Figure 1:**
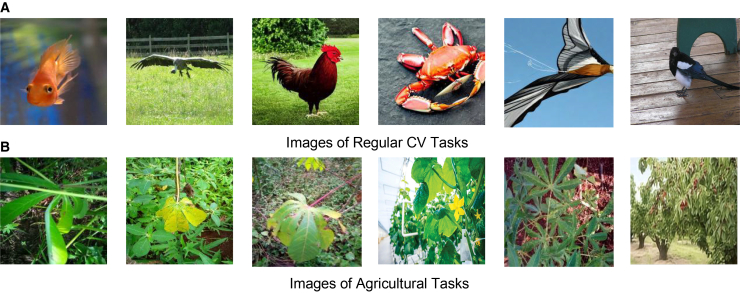
Collect data on two different sets of tasks (A) Images from regular computer vision tasks. (B) Images from agricultural tasks.

Compared to white-box attacks that require full access to model parameters and gradients, patch attacks are more practical for real-world scenarios.^30^^,^^31^^,^^32^ In agricultural disease detection, images are often captured in uncontrolled environments using drones, mobile devices, or field robots.^33^^,^^34^ As a result, they contain complex visual patterns such as fine-grained leaf textures, soil noise, and lighting variations. These characteristics create opportunities for attackers to introduce camouflaged stickers or patch-like objects onto plants or camera lenses. Unlike the clean and structured scenes common in many computer vision benchmarks, agricultural imagery is dominated by irregular shapes, diverse colors, and cluttered backgrounds (see Figure 1), making physical patches harder to detect. For instance, a sticker designed to resemble a natural leaf occlusion could easily mislead plant disease recognition models deployed on UAVs or farm robots.^35^ The open and unstructured nature of farm fields further increases their susceptibility to localized adversarial attacks.

The study of adversarial robustness is critical for reliable deployment in real-world scenarios. While extensive research has been conducted in domains such as autonomous driving and face recognition,^36^^,^^37^^,^^38^ adversarial patch attacks in agricultural disease detection remain underexplored. This creates an urgent need to evaluate model robustness in realistic agricultural environments and to develop attacks aligned with domain-specific visual characteristics. Advancing this direction is crucial for building trustworthy AI systems in precision agriculture.

This study examines whether adversarial patch attacks can be adapted to plant disease recognition by jointly using ViT specific attention behavior and agricultural visual priors. The motivation comes from a domain mismatch. In ViT based attacks, patch locations are often selected according to attention scores. This strategy can be unstable in agricultural images, because high attention may appear on leaf boundaries, background clutter, or visually heterogeneous areas rather than truly disease-related regions.

Patch attacks developed on generic vision benchmarks often transfer poorly to plant disease images, where green-dominant backgrounds, small lesions, irregular leaf structures, and class imbalance make effective patch localization more difficult. To address these challenges, we propose AgroPatch-AutoStrike (APA), an adversarial patch attack framework tailored for ViT-based plant disease detection. Unlike prior methods that rely only on attention scores to determine patch placement, APA incorporates two complementary components to improve attack performance. First, the variance-driven feature patch attack (VDFPA) combines attention scores with intra-patch feature variance and texture-based priors to more precisely identify vulnerable regions. Second, the adversarial focal loss (AFL) adaptively re-weights gradients to mitigate class imbalance such that it can focus on classes that are more resistant to attack. By integrating these two modules, APA strives to achieve more accurate patch placement and higher attack success rates (ASRs).

In our experiments, we tested the injection of one to four patches and systematically studied how patch quantity and location affect model robustness. APA consistently reduces robust accuracy (RA) across multiple ViT architectures, including ViT-S,^39^ SimpleViT,^40^ DeiT,^41^ and DeepViT.^42^ We further evaluated APA’s cross-model transferability and explored misclassification behavior. These results demonstrate that APA is not only effective but also transferable under realistic agricultural conditions, offering new insights into adversarial vulnerabilities in this domain. Our contributions are summarized later in discussion.

• We propose APA, a domain-specific adversarial patch framework tailored for ViT models in agricultural disease detection. APA is also transferable to CNN-based models.
• We develop VDFPA strategy, which integrates attention scores, intra-patch variance, and domain-specific texture cues to accurately identify vulnerable regions. We also propose AFL, which addresses class imbalance by adaptively adjusting gradient contributions based on the robustness of each category.
• We conduct extensive experiments on ViT models using the cassava leaf dataset. Our results demonstrate that carefully placed patch attacks remain a practical threat to self-attention architectures in the agricultural field.

### Related work

Agricultural disease recognition and classification systems have been proposed within the domain of precision agriculture. These systems often utilize deep neural networks to detect and analyze agricultural diseases. In recent years, adversarial attacks on deep neural networks have been an emerging and active research area in machine learning. Existing attack strategies typically generate undetectable attack samples by global perturbation, with the L_P norm used to limit the strength of these changes. In contrast to these methods, adversarial attacks on ViT model^39^ typically focus on patch perturbations rather than total graph perturbations. In this section, we give a short overview of how Transformer models with attention mechanisms are used in agricultural disease detection. We then discuss previous research on ViT from the perspective of adversarial and patch attacks.

### VISION transformers in agricultural disease detection

Given the recent success of self-attentive architectures^20^^,^^43^^,^^44^ on natural language processing (NLP) tasks, the ViT model has emerged as an effective alternative to CNNs in the computer vision field.^45^ The experiments have shown that the ViT-based models outperformed the state-of-the-art deep neural networks (DNNs), such as residual networks (ResNets), inception networks, and EfficientNets, particularly on large-scale datasets such as JFT-300M.^46^ They also achieved higher categorization accuracy on popular datasets, e.g., ImageNet,^47^ CIFAR-100^48^ and Oxford-IIIT Pets.^49^

The self-attention mechanism of ViT has also been adopted for plant disease detection tasks. Fu et al. utilized the patch encoding method of ViT to extract spatial features from apple and grape leaf data, achieving promising results in agricultural disease detection.^50^ To enhance multi-scale feature extraction, Lu et al. incorporated a ghost module into the ViT encoder, leading to a 98.14% accuracy in grape leaf disease detection.^51^ Similarly, Yu et al. demonstrated that integrating inception blocks into the ViT encoder improved local feature extraction, significantly enhancing performance on four common plant disease datasets.^52^ Rachman et al. showed that ViT-based models outperform traditional CNNs, particularly in complex tasks such as rice disease detection, achieving superior recall (97.92%), precision (98.15%), and accuracy (97.92%).^53^ These results consistently surpass models such as visual geometry group (VGG), MobileNet, and EfficientNet, underscoring the effectiveness of ViTs in agricultural disease detection.

### Robustness of ViT

With the wide adoption of ViT, the robustness of the model has also attracted the attention of researchers.^28^^,^^31^^,^^54^ It has been shown that ViT exhibits strong robustness to natural perturbations (based on background noise)^28^^,^^55^ but is more vulnerable than CNN to physical domain adversarial attacks.^25^^,^^56^ Fu et al.^26^ developed patch-fool to attack ViT’s self-attention mechanism and found that ViT’s ability to learn in this setting is weaker than CNNs. However, these studies mainly focused on publicly available datasets and explored the robustness of ViT when the entire image is subjected to natural corruption or adversarial perturbations.

The antagonistic migratory properties of ViT have also been investigated. Mahmood et al.^27^ showed that adversarial samples are not easily migrated between CNNs and transformers, while Naseer et al.^57^ and Yang et al.^58^ proposed techniques to improve adversarial mobility from ViTs to CNNs. Meanwhile, Mao et al.^59^ improved the architectural design of ViTs to enhance robustness. More recently, Chen et al.^60^ demonstrated that ViT’s patch-based representation is inherently more sensitive to localized adversarial patches, prompting new defenses.

### Adversarial attacks

Adversarial attacks^61^ are specially designed small perturbations to the input data, which target confusing AI inference models and leading to wrong outputs. The perturbed inputs are almost indistinguishable from the normal ones. Adversarial attacks are categorized into white-box and black-box attacks based on the attacker’s access to the target model information. In a white-box attack, the attacker has full access to the model architecture and parameters and uses the gradient information of the model loss function to generate adversarial perturbations.^61^^,^^62^ In a black-box attack, the attacker can only access the output of the model and realizes the attack by estimating the gradient of the loss function or using heuristic methods.^63^^,^^64^ These attack methods are of great significance in evaluating and improving the robustness of DNNs. Luo et al.,^9^ You et al.,^10^ and Zhao et al.^65^ explored the vulnerabilities of deep learning models used in plant disease identification under adversarial attacks. They investigated the effect of various white-box attacks on models trained for plant disease identification, showing that even small perturbations can significantly degrade the performance of DNN models. In addition, Li et al.^66^ provided a survey on adversarial attacks in computer vision tasks, summarizing various attack and defense techniques across classification and detection domains. The authors emphasized the need to improve transferability, enhance black-box attack efficiency, and expand adversarial research beyond traditional tasks such as object detection and image classification. These challenges highlight the importance of exploring more diverse and practical scenarios, such as agriculture, where the visual patterns are distinct, and the risks of adversarial misclassification are critical.

### Patch attacks

Unlike traditional adversarial attacks that perturb the entire image, adversarial patching is a specialized attack that limits perturbations to a local region, e.g., pixels within a rectangular area.^67^^,^^68^^,^^69^ The perturbations, while being visible to the human eye, can mislead models in vision-based tasks. Patch attacks are not only the easiest physically implementable adversarial attacks, e.g., putting stickers on traffic signs, wearing specially designed glasses or T-shirts, but also evasive attacks that do not require access to training data. They have become more popular in adversarial attacks in recent years.^70^^,^^71^

The concept of adversarial patching was introduced by Brown et al.,^72^ who proposed a generic and targeted attack against real-world physical objects, laying the foundation for subsequent adversarial patching research. Patch attacks are typically computed using the projected gradient descent (PGD) algorithm, which trains adversarial patches by repeating the gradient descent multiple times to maximize model loss and change the output. We denote the adversarial patch as *X*_*P*_(0), where the subscript *p* ∈ 1, …, *n* denotes the location of the target patch. For the intermediate layer (*L* > 0), the adversarial patch *X*_*P*_(*l*) ∈ *R*^*d*^ is the *p*^*th*^ patch out of *n* patches. Karmon et al.^73^ proposed locally visible adversarial noise (LaVAN), which is smaller than adversarial patches with similar attack effects and focuses more on model weaknesses that lead to misclassification. LaVAN is the first algorithm to introduce steganographic attributes in a patch attack. Along this direction, many other methods have been proposed,^74^^,^^75^ to achieve more practical and effective patch attacks, or to test real-world AI-based software systems under adversarial patches. The state-of-the-art patch-fool^26^ and give me-your-attention (GMYA)^29^ enhance the patch attack by redesigning the loss function. Both schemes integrate scores into losses and maximize the sum of the scores between any two tokens. Both successfully degrade the RA of all ViT/DeiT models to 0% within five 16 × 16 pixel patches.

## Results

Based on the proposed attack framework and the improved loss function, we conducted a comprehensive set of experiments to evaluate the attack’s effectiveness, generalizability, and practical viability in agricultural disease detection tasks. First, the experimental settings of the paper, including materials, performance evaluation metrics, and implementation details, were presented in the experimental setup. In the robustness analysis, the robustness of DNN models under adversarial attacks was evaluated. In the ablation studies, ablation experiments of the paper were conducted, and the performance advantages of the proposed loss function over other loss functions were verified. In the APA evaluation, a series of evaluation experiments were conducted on the APA framework. We compare different patch selection strategies and attack methods. We further assess APA’s transferability and behavioral patterns. Finally, in the visualization analysis, the effectiveness of the proposed method in agricultural disease classification was verified by visualization techniques such as visual attention heatmaps.

### Experimental setup

#### Dataset

We use the cassava leaf disease dataset from the Kaggle 2021 competition. Cassava is a major food crop widely cultivated in tropical regions, including Africa, South Asia, and South America.^76^ It is an important carbohydrate source for many populations and is commonly processed into products such as garri and cassava flour.^77^^,^^78^

Cassava plants are affected by several common leaf diseases. Cassava bacterial blight (CBB) often appears as dark green water-soaked lesions and leaf blight. Cassava brown streak disease (CBSD) is usually associated with chlorotic patches along the veins and brown streak symptoms. Cassava mosaic disease (CMD) causes mosaic such as yellow and green patterns on leaves. Cassava green mite (CGM) damage is commonly reflected by faint yellow green patterns, leaf distortion, and deformed margins.^79^ These symptoms differ in color, shape, and spatial distribution, making cassava disease recognition a challenging fine-grained visual classification task.

**Figure 2:**
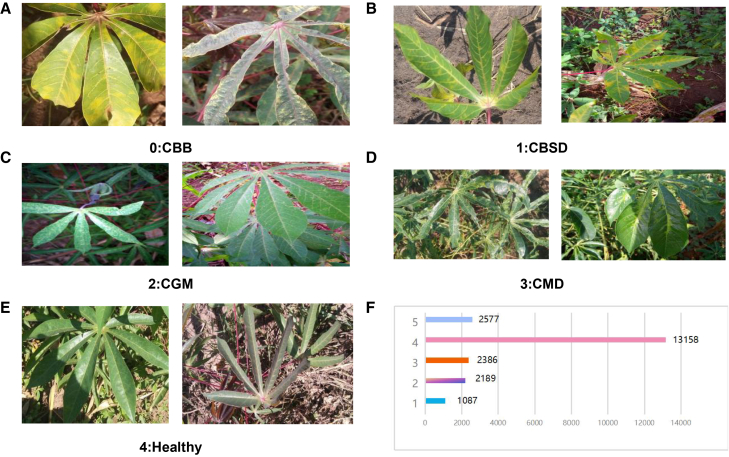
Images of healthy and unhealthy cassava diseases (A) CBB, (B) CBSD, (C) CGM, (D) CMD, (E) Healthy, (F) Quantity.

The dataset contains 21,397 annotated images collected during field surveys in Uganda. Most images were captured by farmers in real garden environments. The labels were annotated by experts from the National Crop Resources Research Institute (NaCRRI) and the Artificial Intelligence Lab at Makerere University in Kampala. The dataset includes five categories: CBB, CBSD, CMD, CGM, and healthy leaves. Figure 2 shows representative examples of diseased and healthy cassava leaves.

The number of images in each category is shown in Table 1. The dataset exhibits a clear class imbalance, with CMD accounting for the largest proportion of samples, while several other categories contain substantially fewer images. This nonuniform distribution reflects practical agricultural data conditions and motivates class-wise robustness analysis in the following experiments.

**Table 1:** Cassava leaf dataset

Label no.	Category	Train	Test	Total
0	CBB	869	218	1087
1	CBSD	1750	438	2189
2	CGM	1909	477	2386
3	CMD	10,527	2631	13,158
4	Healthy	2061	516	2577
Total	–	17,117	4280	21,397

All photos are in RGB format and of the same size (800 × 600 pixels). We randomly crop the original 800 × 600 images to get 224 × 224 sub-images as input to ViT to reduce the complexity of the computational model. Specifically, P is set to 16 in the image preprocessing; that is, the input image is divided into 14 × 14 (i.e., 196) patches before passing through the transformer encoder. This work uses normalization and standardization in the preprocessing stage to ensure that each input pixel has a similar distribution, thus speeding up the convergence of the model. For this reason, we normalize the pixel values to a range from 0 to 1, which helps solve the gradient propagation problem. Normalization improves the stability of gradient propagation and speeds up the training of ViT models. This is done by rescaling the pixel values by multiplying them by 1/255.

#### Threat models

We use models such as ViT-B^39^ and simple-ViT,^40^ DeiT^41^ and deep-ViT^42^ to validate the effectiveness of our proposed method. To further validate the generalization of the method, we introduce CNN-based models. The ResNet and ViT models from the paper and some of their training settings are listed in Table 2. We used the cassava disease dataset, which provides a total of 21,397 images, to evaluate the classification accuracy of the models. The input size for training was *H* = *W* = 224, and the patch size was set to 16, for a total of 196 patches per image. The accuracy of clean images is shown in Table 2, where the accuracy of ViT on clean images is better than the corresponding CNN model.

**Table 2:** Comparison of popular CNN and ViT models

Model	Params	ViT backbone layers Hidden size	Clean Acc	Patch size	Input size
ResNet-18	23M	–	0.7314	–	224∗224
ResNet-34	52M	–	0.718	–	224∗224
ResNet-50	98M	–	0.7347	–	224∗224
VGG-16	134M	–	0.7582	–	224∗224
ViT-B	86M	12/768	0.8510	16∗16	224∗224
simple-ViT	22M	12/384	0.7495	16∗16	224∗224
Deit	86M	12/768	0.7110	16∗16	224∗224
ViT-S	22M	12/384	0.7345	16∗16	224∗224
DeepViT	27M	12/768	0.7058	16∗16	224∗224
Vit-tiny	21M	12/192	0.7385	16∗16	224∗224

#### Attack methods

We follow the APA approach to generate adversarial patch attacks. Images that were correctly classified were first randomly selected from the cassava validation set. Each reported RA score is averaged over all validation set images. We used five attack methods (i.e., fast gradient sign method (FGSM),^80^ PGD,^61^ basic iterative method (BIM),^81^ patch-fool^26^ and LaVAN^73^) to evaluate the robustness of the model. For baseline patch attacks, the patch size is set to 16 × 16 pixels unless otherwise stated. For APA, patch locations are determined by the proposed localization strategy rather than being fixed at a predefined position. Table 3 presents a structural comparison between our proposed APA framework and two representative patch-based attacks, patch-fool and LaVAN, highlighting the architectural innovations and transferability of APA in agricultural scenarios.

**Table 3:** Conceptual comparison of APA with representative patch attack methods

Method	Type	Placement	Mechanism	Transfer
Patch-fool	untargeted	attention	attention and gradient	limited
LaVAN	targeted	gradient	mask and loss	no
APA (Ours)	untargeted	attention and feature	AFL	cross-model (ViT/CNN)

#### Attack setup

In the proposed APA attack, perturbations are restricted to one or more local patch regions rather than being applied to the whole image. We focus on a model-guided digital patch setting for robustness evaluation. APA follows an untargeted attack setting. Its objective is to reduce the overall classification accuracy of the disease recognition model, rather than force predictions into a predefined target class. Pixels outside the selected patch regions remain unchanged. The patch localization process uses model-side information, including gradients and attention maps, to identify vulnerable local regions. This setting provides a diagnostic evaluation of model robustness under strong attack access.

For all patch-based attacks, each patch has a size of 16 × 16 pixels unless otherwise stated. For DeiT-S^44^ with 14 × 14 input tokens, one patch corresponds to approximately 1/196 of the input token area. This setting keeps the perturbation spatially localized and separates patch attacks from full image perturbation attacks.

In the improved attention score equation, the weighting coefficient *λ* is set to 8. In the VDFPA scoring equation, the weighting coefficient *β* is set to 0.2, and in the AFL equation, the focusing parameter *γ* is set to 1. The effects of these parameters are reported in the ablation studies. To evaluate the performance of APAs under different perturbation strengths, we allow APAs to choose up to four patches to attack based on the attack framework presented in the variance-aware patch attack (VAPA) and agri-feature patch optimization (AFPO) sections, i.e., Select patches in descending order based on the scores in the VDFPA scoring formula. Note that in this paper, we report robustness accuracy rather than attack success, as our main focus is on the robustness of the disease classification model.

To train the distributed mapping network, we use the Adam optimizer with an initial learning rate *e* of 1e−4 and perform 100 training cycles. The learning rate is attenuated by a factor of 0.2 every $\frac{1}{1+{\text{Item}}_{i}}$ cycles. The attack step size and the number of iterations are set to 0.2 and 200, respectively.

#### Evaluation metric

We use (1) RA to evaluate the robustness of the model. RA is an important measure of the model’s ability to maintain correct predictions in the face of adversarial samples. A set of correctly categorized images is collected, denoted as *N*, which is also the number of adversarial samples. *C* is denoted as the number of correct predictions made by the model on the adversarial samples after the attack. Define $\text{RA}=\frac{C}{N}$. The higher the RA value, the higher the robustness of the model; (2) ASR to evaluate the effectiveness of adversarial attacks. ASR measures the model’s susceptibility to adversarial samples. Given a set of adversarial samples *N*, where *S* represents the number of samples for which the attack successfully alters the model’s prediction. Define $\text{ASR}=\frac{S}{N}$. A higher ASR indicates a more successful attack and lower model robustness. All experiments are conducted on the full set of correctly classified validation samples, without random subsampling. Under this evaluation protocol, the reported RA and ASR values are determined by the fixed validation set and attack configuration.

#### Experimental platform

The experimental platform used in this study: the hardware environment is a 12th Gen Intel(R) Core(TM) i7-12700KF CPU 3.60 GHz, 64G RAM, NVIDIA GeForce RTX 4070 Ti SUPER GPU; the software environment is a Windows 11 system, Python3.8, PyTorch 2.3.0.

### Robustness analysis of DNN models under adversarial attacks

In this section, we evaluated the performance of various models in agricultural disease recognition. The results confirmed that ViT models excel in handling complex agricultural background images. We further investigated the effects of two types of attacks on the robustness of ViT models in real agricultural scenarios. The first category is white-box attacks,^82^ including common gradient-based adversarial attack methods such as FGSM,^80^ PGD,^61^ and BIM.^81^ The second category is patch attacks,^72^ including adversarial patch attacks such as token-attack, patch-fool, and GMYA. To facilitate comparison with white-box attacks, we also use gradient based optimization to implement the patch attack. The experimental results were unexpected. The attack effects of the white-box attack and the patch attack in real agricultural scenes were relatively limited, especially since the patch attack could hardly significantly reduce the accuracy of the model, as shown in Table 4. It compares the robustness of various CNN and ViT models on real-world agricultural data under white-box and patch attacks. The results indicate that ViTs, such as ViT-B and simple-ViT, generally achieve higher clean accuracy than CNNs (e.g., ResNet-18, ResNet-50). Under adversarial attacks, ViTs demonstrate stronger robustness, particularly ViT-B, which maintains relatively high RA across most attack types and shows notable resilience against gradient-based patch attacks. In contrast, ResNet models exhibit lower RA under patch attacks, underscoring the superior resilience of ViT models in adversarial scenarios on agricultural datasets. These findings reveal the limitations of existing attack methods in dealing with agricultural disease images in real-world scenes.

**Table 4:** Results of white-box and patch attacks on real-world agricultural data (Evaluate the RA of ViT and CNN models under white-box attacks: FGSM, PGD, BIM; patch attacks: random, gradient on the cassava dataset)

Model	Clean ACC	RA				
		FGSM	BIM	PGD	Random (Patch)	Gradient (Patch)
ResNet-18	0.7314	0.5975	0.4635	0.5044	0.5733	0.2554
ResNet-50	0.7347	0.5066	0.5679	0.5665	0.569	0.4055
VGG-16	0.7582	0.6393	0.701	0.8172	0.6962	0.6208
ViT-B	0.851	0.6584	0.7444	0.5714	0.7178	0.6972
Simple-ViT	0.7495	0.4949	0.6046	0.3221	0.4269	0.4424
Deit	0.711	0.6067	0.6262	0.5731	0.5679	0.5449

Further investigation revealed that the attack result differed significantly for different patch positions (as shown in Table 4. Therefore, we designed targeted patch attacks tailored to the structure of DNN models, particularly ViT models, leveraging their attention mechanisms and the characteristics of agricultural image data.

### Ablation studies

To evaluate the contribution of the proposed patch localization components, we conduct two groups of ablation experiments on the cassava leaf disease dataset. The first group analyzes the internal design of the variance-aware patch attack (VAPA), with a focus on the role of attention scores and intra-patch feature variance in patch selection. The second group evaluates the two submodules of VDFPA, VAPA, and AFPO to examine their individual and joint effects on adversarial patch localization. All ablation experiments use the same attack budget, patch size, model settings, and loss function. RA is used as the evaluation metric, where a lower RA indicates a stronger attack effect. Clean accuracy is also reported to show that the underlying classification performance of each model remains unchanged across different attack settings.

### Ablation study of variance aware patch selection

Table 5 reports the ablation results for VAPA. We compare four patch selection strategies under the same attack setting: gradient-based selection, attention-only selection, variance-only selection, and VAPA. The gradient-based method serves as the baseline. The attention-only and variance-only settings are used to isolate the effects of attention scores and intra-patch feature variance, respectively, while VAPA evaluates their combined effect.

**Table 5:** Ablation study of variance aware patch selection in VAPA

Patch selection setting		DeepViT		ViT-B		ViT-S	
Attention Score	Variance	RA	Clean Acc	RA	Clean Acc	RA	Clean Acc
X	X	0.4692	0.7058	0.7001	0.8510	0.3070	0.7345
✓	X	0.4545	0.7058	0.7013	0.8510	0.2985	0.7345
X	✓	0.4724	0.7058	0.7185	0.8510	0.3064	0.7345
✓	✓	0.4312	0.7058	0.6892	0.8510	0.2904	0.7345

As shown in Table 5, both attention-only and variance-only selection generally reduce RA compared with the gradient-based baseline. Their combination in VAPA yields the strongest attack performance across the three ViT models. On DeepViT, RA decreases from 0.4692 with gradient-based selection to 0.4545 with attention-only selection and 0.4724 with variance-only selection. VAPA further reduces RA to 0.4312. Similar improvements are observed on ViT-B and ViT-S.

These results suggest that feature variance provides information complementary to attention scores. Attention scores identify model-sensitive regions, whereas feature variance favors internally coherent plant regions and suppresses visually unstable patches. Their combination improves patch localization in agricultural disease images.

**Figure 3:**
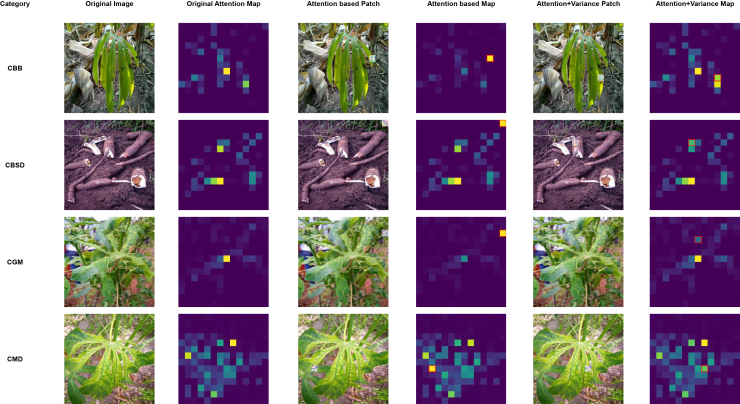
Visualization of patch selection with attention variance guidance

Figure 3 presents patch locations selected with and without variance guidance. Each row corresponds to a disease category. The columns show the original image, the clean attention map, the attention-based patch result and its attention map, and the variance-guided patch result and its attention map. The results show that attention-based selection may focus on highly activated but visually unstable regions. After incorporating feature variance, the selected patch locations tend to shift toward more coherent plant regions. This observation is consistent with the quantitative results in Table 5.

### Module wise ablation study of VDFPA

We further evaluate the contribution of VAPA and AFPO within the complete VDFPA module. The experiments are conducted based on the gradient-based strategy, with the serves attack method as the baseline. We conducted an ablation experiment on the proposed attack framework on the cassava dataset.

The results in Table 6, show that both our proposed VAPA and AFPO modules can improve the attack effectiveness of the model compared to the baseline approach. Especially for the VAPA module, on ViT-B, the RA of the model decreases from 85% to 68% in the alignment setting. On ViT-S, VAPA alone achieves the lowest RA of 0.2852, while VDFPA obtains a comparable RA of 0.2887. This result suggests that VAPA is the dominant contributor on ViT-S, whereas AFPO provides clearer complementary gains on DeepViT and ViT-B. Overall, VAPA and AFPO provide complementary benefits for adversarial patch localization. The combination of our proposed VAPA and AFPO, the complete attack method can further effectively improve the attack strength of agricultural disease models in the face of patch attacks.

**Table 6:** Ablation study of our proposed VDFPA method

Setting		VIT		VIT-B		VIT-S	
VAPA	AFPO	RA	Clean Acc	RA	Clean Acc	RA	Clean Acc
X	X	0.4537	0.7058	0.6949	0.8510	0.3016	0.7345
X	✓	0.4290	0.7058	0.6978	0.8510	0.2939	0.7345
✓	X	0.4312	0.7058	0.6892	0.8510	0.2904	0.7345
✓	✓	0.4280	0.7058	0.6773	0.8510	0.2887	0.7345

### The impact of the hyperparameter

To control computational cost and ensure clarity of interpretation, we employed a one-dimensional sweep strategy during hyperparameter tuning. That is, each parameter varied individually while holding others fixed. This allows us to isolate the impact of each hyperparameter on adversarial performance.

#### Effect of different *λ* values

**Figure 4:**
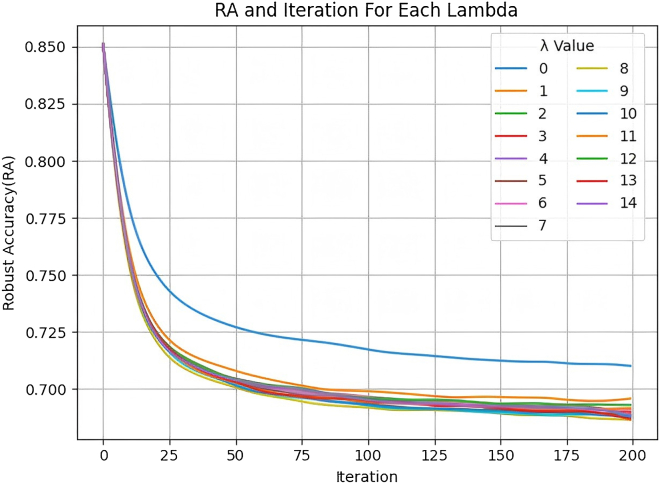
Effect of different λ values on model RA over iterations Higher λ values intensify the variance constraint term in the adjusted attention score.

For the optimization process of VAPA, we conducted experiments to evaluate the impact of the balancing coefficient *λ* in the adjusted attention score. The term $\left(1-\sigma_{i,j}^{2}\right)$ reflects color-texture stability, typically close to 1 in leaf regions where variance is low. Because $\sigma_{i,j}^{2}$ usually ranges between 0 and 0.05 in our dataset, a larger *λ* is required to adequately scale its influence on *A*_*i*_. We therefore varied *λ* in a wider range, from 0 to 14, to study its effect on attack performance. Figure 4 shows the changes in RA across iterations under different *λ* values. A smaller *λ* results in minor attention adjustment, while a larger *λ* places stronger emphasis on variance modulation, which suppresses unstable regions more aggressively. Empirical results suggest that when *λ* = 8, the VAPA module achieves the best trade-off between attention fidelity and patch semantic stability, yielding optimal patch location selection and improved attack effectiveness. These observations support our design choice of including the variance-driven reweighting mechanism in the scoring function.

#### Effect of different β values

To realize the effective attack localization search process based on two modules (VDFPA), we conducted experiments. By assigning weight information to the proposed module, we study the corresponding attack effect under different weight values. As shown in Table 7, we have plotted the RA of each model under different *β* values. The experimental results show that when *β* = 0.2, the optimal patch location search can be achieved by VDFPA.

**Table 7:** Effect of different *β* values

*β*	RA	Clean Acc
0.2	0.675	0.851
0.5	0.676	0.851
0.8	0.676	0.851
1.0	0.676	0.851
1.5	0.678	0.851
2.0	0.679	0.851
2.5	0.680	0.851
3.0	0.684	0.851

#### Effect of different $\alpha_{i}^{*}$ and γ values

In our design of the adversarial focus loss function, there are two hyperparameters, $\alpha_{i}^{*}$ and *γ*. To investigate their optimal settings through experimental analysis, we implemented the weighted cross entropy loss as described in the adversarial focal loss (AFL) section. The experimental outcomes for varying values of *α* are displayed in Table 8. Among them, *a*_*i*_ represents setting α to the reciprocal of frequency, and $\alpha_{i}^{*}$ represents when *α* is normalized. Optimal attack performance was achieved when *α* values were set as normalized.

**Table 8:** Effect of different *a* values

*a*	RA	Clean Acc
*a* = none	0.6972	0.851
*a* = *α*_*i*_	0.6943	0.851
$a=\alpha_{i}^{*}$	0.6831	0.851

The results using our proposed adversarial focusing loss are shown in Table 9. The adversarial focusing loss introduces a new hyperparameter, the focus parameter *γ*, which is responsible for controlling the strength of the modulation term. When *γ* = 0, our loss function is equivalent to the cross-entropy loss, and as *γ* increases, there is some increase in AFL over CE. Using *γ* = 1, AFL reduces the RA by 1% over a balanced CE loss. The experimental results show that the model achieves the best attack when *α* is the inverse of the normalized frequency of each category and *γ* = 1.

**Table 9:** Effect of different *γ* values

*γ*	RA	Clean Acc
0	0.69	0.85
0.5	0.83	0.85
1	0.66	0.85
2	0.82	0.85
5	0.67	0.85

#### Effect of different l values

We also performed an ablation study in the improved attention mechanism section to determine the layer *l* for adversarial patch selection. As shown in Table 10, choosing later layers generally produces more consistent results than choosing earlier layers. We speculate that this is because as the number of layers increases, the model mixes information more fully in the self-attention mechanism. The last layer is able to integrate global information and identify more representative and important patches, making adversarial perturbations more effective. This conjecture is verified by conducting adversarial attacks on each layer; that is, the model is most affected by adversarial attacks at layer 12. Therefore, we set *l* = 12 by default.

**Table 10:** Analyze the effect of layer index on adversarial patch position selection

*l*	1	2	3	4	5	6
ViT-B	0.7079	0.7025	0.7091	0.6981	0.6889	0.6850
*L*	7	8	9	10	11	12
ViT-B	0.6906	0.6887	0.6997	0.6945	0.6826	0.6806

The table includes the resulting RA.

### Experimental design verification

#### Feature vector variance

**Figure 5:**
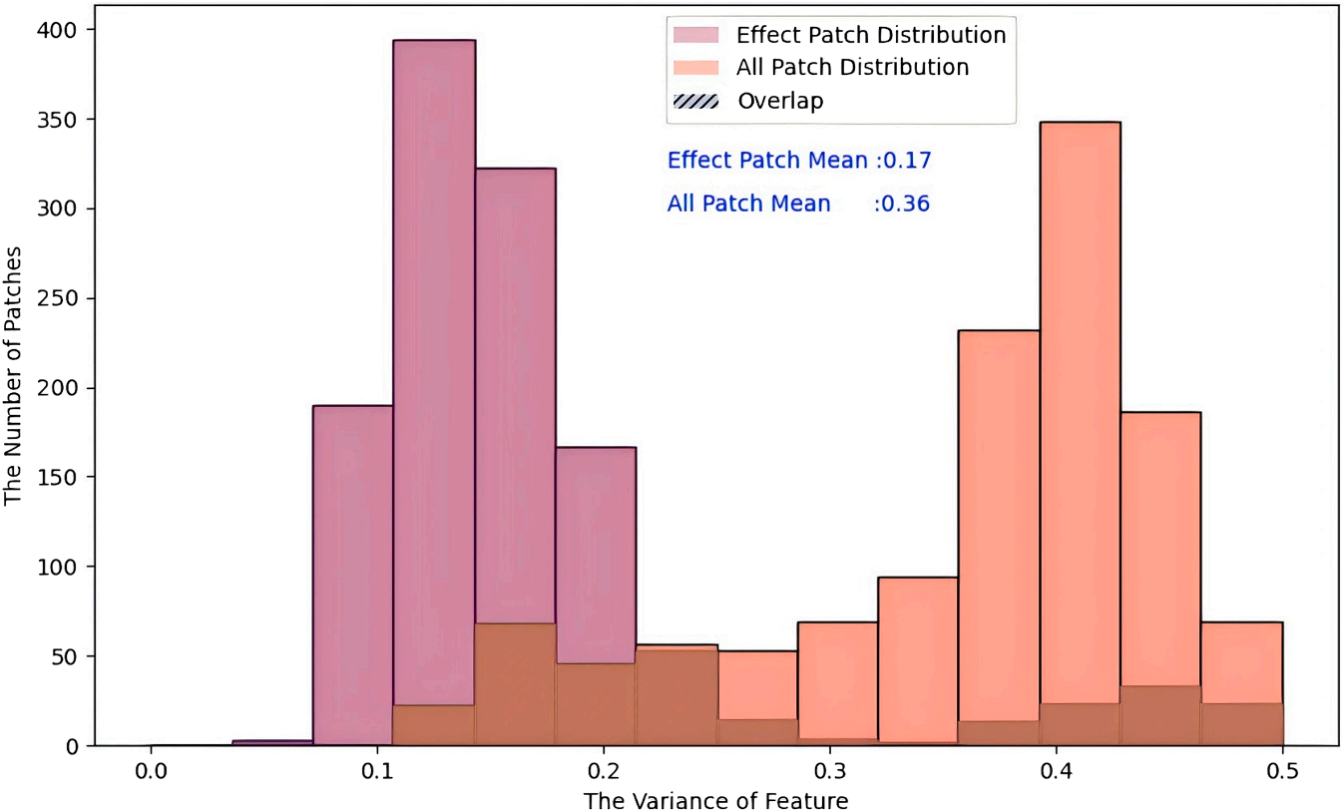
Feature variance of effect patch vs. all patch

As shown in Figure 5, there is a significant difference between the distribution of the effective patch (the patch corresponding to the successful attack) variance (∂_*e*_) and all patch variance ∂_*a*_), where ∂_*e*_ is significantly smaller than ∂_*a*_. The results suggest that feature variance can be used as an effective metric for selecting target patches against attacks. Patches with smaller feature variance are more likely to affect the model’s predictions through attack perturbations, thereby increasing the success rate of adversarial attacks.

**Figure 6:**
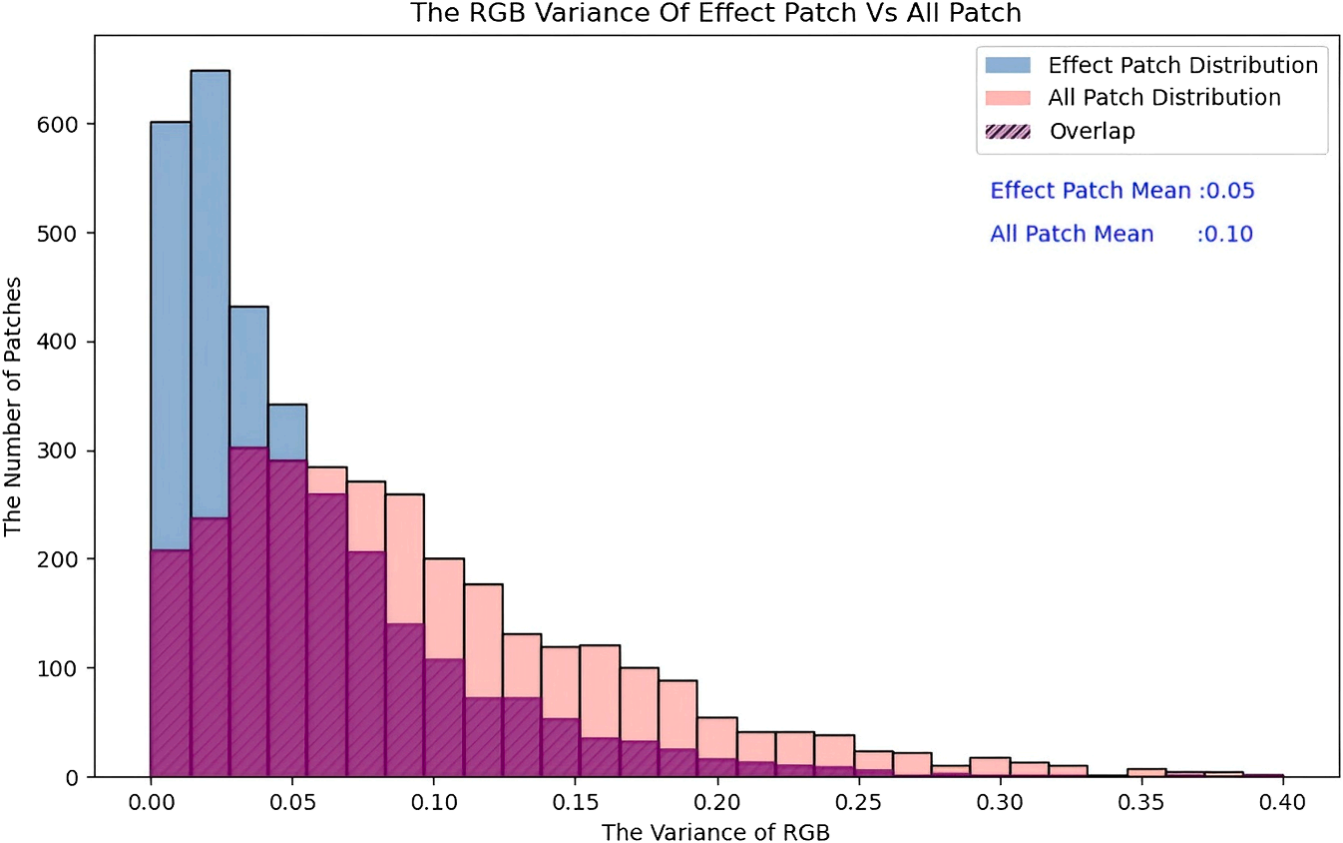
RGB variance distribution of effect patch vs. all patch

Through analysis of the RGB variance between effective patches and all patches across the entire image shown in Figure 6), we observed that the RGB variance of effective patches is significantly concentrated in a lower range, reflecting color consistency and stability. In contrast, the RGB variance of patches across the entire image is more widely distributed, indicating greater variability in color. This result demonstrates the effectiveness of RGB variance as a feature metric. It shows that in agricultural image analysis, using RGB variance can effectively identify and distinguish key patches in adversarial attacks.

#### Green ratio

**Figure 7:**
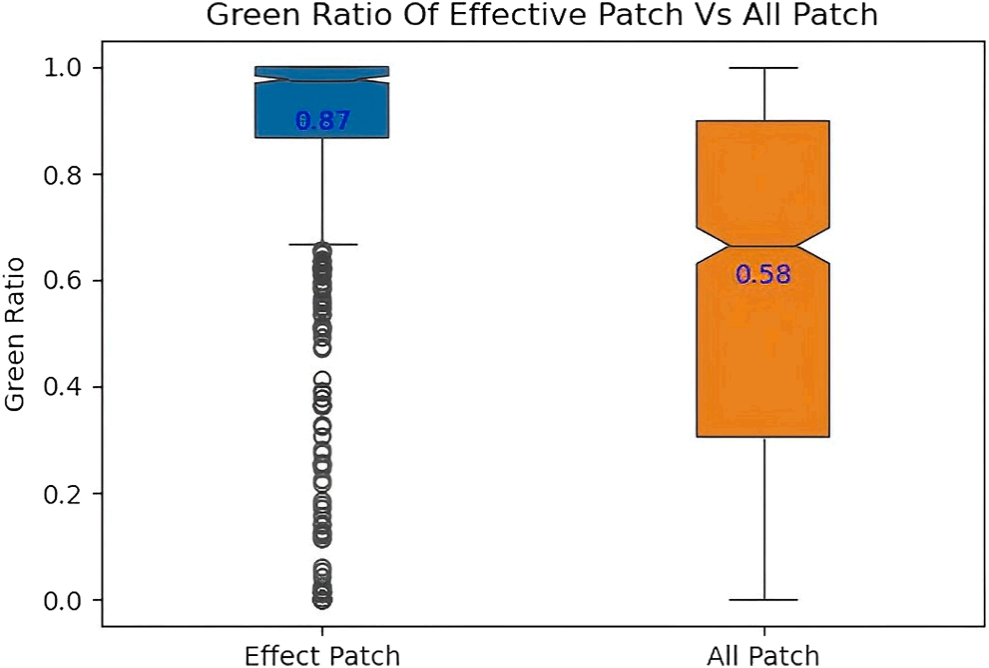
Green ratio distribution of valid patches vs. global patches

An analysis of the green ratio in effective patches compared to patches across the entire image (refer to Figure 7) reveals a significant divergence in the distribution of green ratios between the two groups. Specifically, the green ratio for effective patches predominantly falls between 85% and 100%, while for patches across the entire image, it ranges mostly between 30% and 85%. This result validates the effectiveness of the green ratio as a feature indicator.

#### Similarity assessment

We further validate the effectiveness of the hybrid feature criteria proposed in the similarity assessment section. By comparing the Euclidean distances of the patch feature vectors generated by the AFPO method and the random attack, the results show, as shown in Table 11, that the vector distances of the former are significantly shorter than those of the latter. This shows that the AFPO method is more effective in identifying and selecting key patches, and can significantly improve the success rate and accuracy of adversarial attacks.

**Table 11:** Euclidean distance under different selection methods

Model	Selection strategy	Euclidean distances
VIT-B	Random	0.6438
	AFPO	0.2128

### Attack performance under class imbalance

To examine the impact of class imbalance, we report class-wise RA and ASR under adversarial patch attacks. Table 12 presents the results across different disease categories. The results show clear differences in attack difficulty across disease categories. CMD accounts for the largest proportion of the dataset and maintains the highest RA of 0.8637, with the lowest ASR of 0.1363. In contrast, several smaller categories show lower RA values, indicating higher vulnerability under the evaluated attack setting. This variation suggests that class distribution may influence adversarial robustness in plant disease recognition models. In particular, categories with fewer samples tend to exhibit less stable robustness under localized perturbations. This indicates that class imbalance may interact with model behavior during adversarial attacks. It highlights the importance of considering class-wise robustness when evaluating agricultural disease recognition systems.

**Table 12:** Cassava disease categories: Adversarial attack performance and robustness statistics

Category	Category proportion	Clean ACC	ASR	RA
CBB	0.05	0.5621	0.5948	0.4052
CBSD	0.10	0.7058	0.4788	0.5212
CGM	0.11	0.6852	0.6101	0.3899
CMD	0.61	0.9643	0.1363	0.8637
Healthy	0.12	0.6306	0.8980	0.1020

### Effectiveness of the adversarial-focal loss

To evaluate the effectiveness of our AFL (the adversarial focal loss (AFL) section), we compare it with the baseline: training only with the final cross-entropy loss, i.e., enabling L_ce_ without L_AFL_ as shown in Table 13. We can observe that our proposed focal adversarial loss can achieve the best attack performance; for example, in different ViT models, the model robustness accuracy after patch attack is reduced by 3% and 1%, respectively, compared with the baseline without AFL.

**Table 13:** Benchmark the AFL with baselines based on cross-entropy loss

Model	Loss function	RA			
		1P	2P	3P	4P
ViT-B	L_ce_	0.69	0.57	0.47	0.40
	L_AFL_	0.66	0.55	0.47	0.39
ViT-T	L_ce_	0.37	0.15	0.05	0.01
	L_AFL_	0.36	0.14	0.04	0.01

### Evaluating APA: robustness and effectiveness in agricultural disease detection

To validate the effectiveness of our proposed APA approach, we conducted a series of experiments from multiple perspectives. These include the impact of patch selection and quantity, comparisons with existing attack baselines, transferability, and attack directionality. These experiments comprehensively evaluate the effectiveness, practicality, and potential applicability of APA under real-world agricultural scenarios.

### The impact of patch size in different patch selection strategies

We evaluated the effectiveness of our APA approach by comparing two patch selection methods: (1) random patch selection; (2) attention score-based patch selection. In the latter, the average attention score of each patch on layer 12 was used as the selection metric. For consistency, the experiments utilized the cross-entropy loss *L*_*ce*_ without additional neural components.

In terms of the impact of the number of patches on the effectiveness of the attack, as shown in Table 14, the impact of patch quantity on attack effectiveness is significant. As the number of adversarial patches increases, the robustness of all models consistently decreases, indicating that multi-patch strategies can effectively weaken model defenses. When the attack exceeds 4 patches (about 2% of the input image area), the deception rate of simple-ViT, ViT-S, and DeepViT reaches almost 100%, while ViT-B and DeiT experience a roughly 50% reduction in robustness compared to single-patch attacks, highlighting their heightened sensitivity to multiple patch perturbations.

**Table 14:** RA of ViT and CNN models across various patch selection strategies, where “nP” represents the number of perturbed patches

Model	Select strategy	RA			
		1P	2P	3P	4P
ResNet-18	random	0.4786	–	–	–
	gradient	0.4796	–	–	–
	ours	0.4474	–	–	–
ResNet-34	random	0.5733	–	–	–
	gradient	0.2554	–	–	–
	ours	0.1706	–	–	–
VGG-16	random	0.6962	–	–	–
	gradient	0.6208	–	–	–
	ours	0.2704	–	–	–
ViT-B	random	0.7178	0.5898	0.4908	0.4138
	attention	0.6972	0.5671	0.4688	0.3903
	ours	0.6800	0.5611	0.4626	0.3882
simple-ViT	random	0.4953	0.3059	0.1828	0.0935
	attention	0.4763	0.2676	0.1440	0.0778
	ours	0.4786	0.2657	0.1403	0.0737
Deit	random	0.5704	0.4619	0.3783	0.2885
	attention	0.5564	0.4454	0.3522	0.2741
	ours	0.5587	0.4396	0.3419	0.2500
ViT-S	random	0.3100	0.0943	0.0262	0.0018
	attention	0.3016	0.0869	0.0169	0.0028
	ours	0.2887	0.0778	0.0167	0.0026
DeepViT	random	0.4612	0.2585	0.1151	0.0744
	attention	0.4537	0.2486	0.1396	0.0764
	ours	0.4280	0.2478	0.1369	0.0608

As shown in Table 14, we can see that: (1) APA patch selection proves to be the most effective approach for attacking the three ViTs in most scenarios, making it the default choice; (2) Under similar model complexity, CNN models are less robust to adversarial attacks than ViT models, indicating weaker defense against single-patch attacks. In contrast, ViT models maintain higher robustness even with attention-based or proposed methods, suggesting that the self-attention mechanism helps ViTs better resist single-patch attacks; (3) Using random patch selection, there is a maximum robustness accuracy gap of 4% with the best strategy, which can effectively reduce the performance robustness of ViT-B.

Additionally, this comparative analysis illustrates that APA performs more effectively than existing gradient-based and patch-based attacks across both ViT and CNN architectures. Although designed primarily for ViT models, APA’s strong performance on CNNs further validates its generalization capability across different network backbones. This demonstrates APA’s potential to enhance adversarial attack strategies in diverse model architectures, highlighting its adaptability and robustness.

### Robustness against different attack methods

To further validate the effectiveness of our proposed attack framework, we incorporated various attack methodologies for evaluation, comparing the performance of white-box attacks, state-of-the-art patch attacks, and our proposed APA method.

In order to comprehensively evaluate the attack effectiveness of the proposed APA, we first use the widely used white-box-based attack methods, such as FSGM, BIM, PGD, and some state-of-the-art attack methods designed for the ViT model. For example, patch-fool, LAVAN, and so forth. to evaluate the RA of the model. Through experiments, we detail all the attack methods used. As shown in Table 15. Our proposed APA achieved better attack results in the vast majority of cases under CNN and transformer models, with the simple ViT model having an average RA of 47%. Compared to the baseline, the average RA is reduced by almost 3%. This proves the effectiveness of our proposed APA.

**Table 15:** Robustness against different attack methods

Model	Clean ACC	FGSM	BIM	PGD	Patch-fool	Lavan	ours
ResNet-18	0.7314	0.5975	0.4635	0.5044	–	0.6273	0.4470
ResNet-34	0.718	0.5845	0.4610	0.4798	–	0.6710	0.1706
ResNet-50	0.7347	0.5066	0.5679	0.5665	–	0.6748	0.5650
VGG-16	0.7582	0.6393	0.7010	0.6177	–	0.7408	0.2704
Vit-B	0.851	0.6584	0.7444	0.6960	0.6875	0.6898	0.6773
simple-ViT	0.7495	0.4949	0.6046	0.4813	0.4770	0.5028	0.4710
Deit	0.711	0.6067	0.6262	0.5731	0.5796	0.6047	0.5582
DeepViT	0.7058	0.4591	0.5869	0.4383	0.4438	0.6684	0.4280

### Patch stability under local transformations

To further examine the stability of adversarial patches, we evaluate APA under several patch-level transformations. Unlike full-image corruption settings, the transformations are applied only to the generated patch, while the remaining image content is kept unchanged. This setting simulates mild local appearance variations that may occur during practical deployment.

After generating the adversarial patch and determining its location, we apply three local transformations to the patch. Specifically, the patch is rotated by ±15°, resized by 0.8× or 1.2×, and then cropped or padded back to 16 × 16 pixels, or adjusted in brightness by multiplying the pixel intensity by 0.8 or 1.2. The transformed patch is then pasted back to the original location, and RA is evaluated under the same protocol. Table 16 reports the average RA under each transformation type.

**Table 16:** RA under patch level transformations (RA is averaged over the two settings for each transformation type)

Method	No Transform	Rotation (±15°)	Scaling (0.8×/1.2×)	Brightness (0.8×/1.2×)
Patch-fool	0.6875	0.7388	0.7493	0.6814
LaVAN	0.6898	0.7664	0.7694	0.7020
APA	0.6773	0.7270	0.7153	0.6719

The results show that APA consistently achieves lower RA than patch-fool and LaVAN under all evaluated transformation settings. In addition, APA exhibits smaller RA changes under rotation, scaling, and brightness variation, indicating that its attack effectiveness is less sensitive to local appearance perturbations. These observations suggest that the proposed patch selection strategy provides better local transformation stability under mild physical disturbance conditions.

### Cross-model transferability analysis

To evaluate the cross-model transferability of APA-generated adversarial patches, we conducted experiments using three ViT-based models (ViT-B, ViT-S, and SimpleViT) as source models. The patches generated from each were then transferred to six different target architectures, including both transformer and CNN families. Table 17 presents the RA of each target model under transferred attacks, where a lower RA indicates stronger attack transferability. APA demonstrates effective cross-model transferability, particularly when transferring from ViTs to CNNs.

**Table 17:** Cross model transferability

Source Model →Target Model	ViT-B	ViT-S	Simple-ViT	ResNet-18	ResNet-34	VGG-16
ViT-B	0.6783	0.7265	0.7401	0.5176	0.1717	0.2796
ViT-S	0.8395	0.2927	0.7396	0.5213	0.1711	0.2807
Simple-ViT	0.8472	0.7189	0.4572	0.5197	0.1701	0.2808

Patches generated from SimpleViT, for example, reduce the RA of ResNet-34 to 0.1701 and that of VGG-16 to 0.2808. While intra-ViT transfer (e.g., from ViT-S to ViT-B) results in relatively higher RA values (e.g., 0.8395), cross-architecture transfer yields lower RAs, indicating a stronger adversarial impact. Notably, ResNet-34 consistently shows the lowest RA (0.1701) across all transfer settings, highlighting its particularly weak defense against APA’s cross-model attacks. These results suggest that although APA was tailored for ViT architecture, the adversarial patches it generates exhibit black-box transferability, especially when applied to CNNs. This makes APA not only effective in white-box settings but also practical in realistic adversarial scenarios where model internals are inaccessible.

### Attack directionality and class specific vulnerability

**Figure 8:**
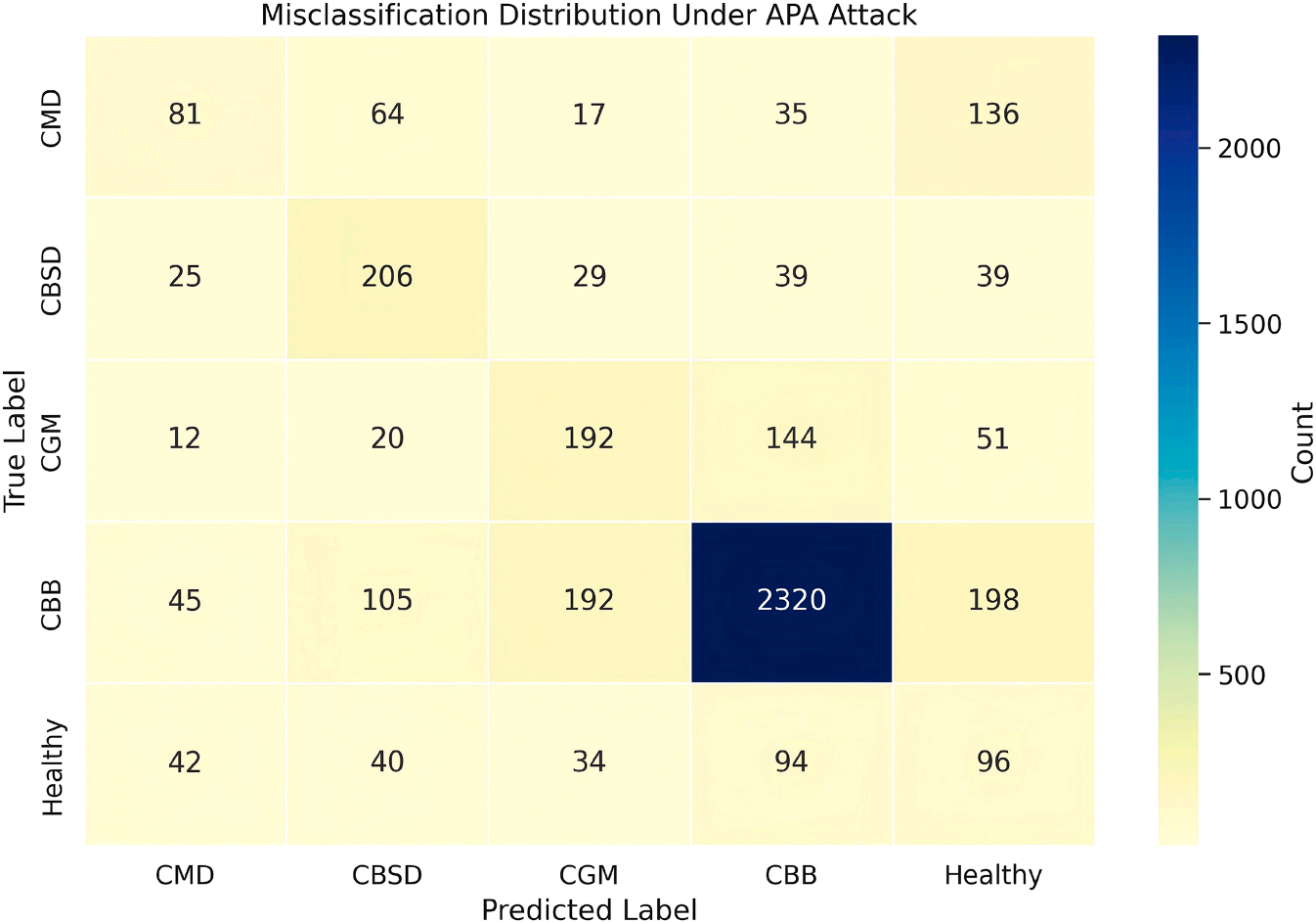
Misclassification distribution under APA attack

To investigate the directional behavior of adversarial attacks, we analyze the misclassification distribution induced by our APA method. Specifically, we use a confusion matrix in Figure 8 to visualize how true labels are transformed into incorrect predictions under attack.

CBB emerges as the most stable class, with the majority of its samples (2320) still correctly predicted even under attack. This suggests strong model confidence and robustness toward this class. In contrast, CMD shows high directional vulnerability, with 136 instances being misclassified as healthy and 64 misclassified as CBSD. This indicates that the APA perturbation tends to shift CMD predictions toward non-disease classes. CGM is highly prone to being misclassified as CBB, with 144 mispredictions, suggesting that APA easily exploits overlapping visual features between CGM and CBB. These observations reveal that APA exhibits class-specific directional tendencies rather than purely random misclassification. Asymmetric behavior implies that certain classes act as dominant attractors during adversarial attacks, either due to visual similarities or model biases. This insight provides a deeper understanding of the attack dynamics and validates the need for direction-aware robustness evaluations in agricultural disease recognition.

### Visualization

In this Section, visualization methods are employed to further validate the effectiveness of the attack framework proposed in this paper.

**Figure 9:**
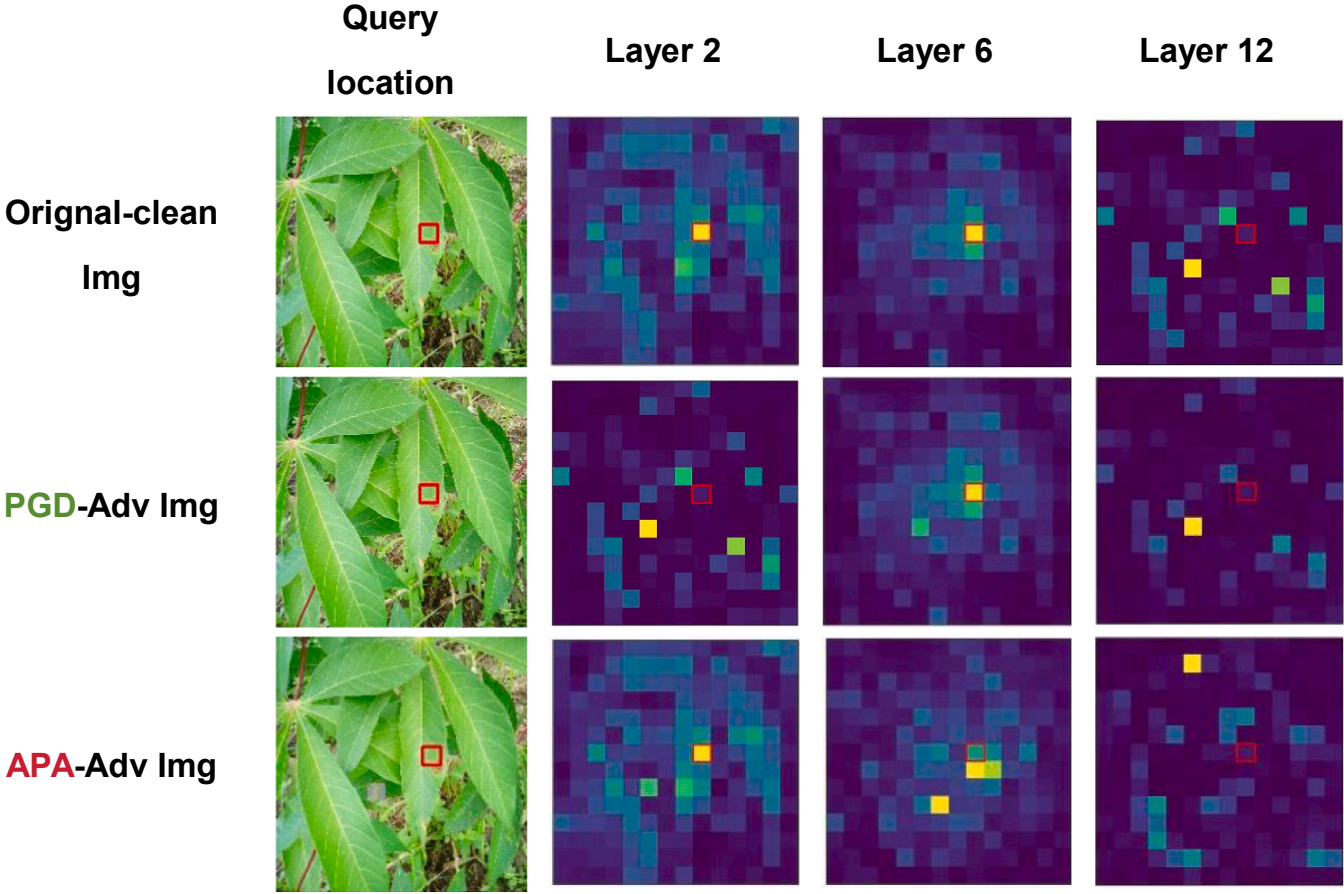
Comparison of attention map disparities in ViT-B under clean, PGD, and APA attacks

Figure 9 compares attention maps for clean inputs, adversarial inputs generated by the PGD attack *ϵ* = 1/255, and those produced by the APA attack in the intermediate and final layers of ViT-B. Attention scores are averaged across heads per layer, showing token scores relative to the query token (center patch marked by a red box in this case study). The attention maps for clean and PGD adversarial inputs show minimal differences across layers. In comparison, the APA attack significantly widens the difference between clean and adversarial attention maps, highlighting its effectiveness in compromising ViT models.

**Figure 10:**
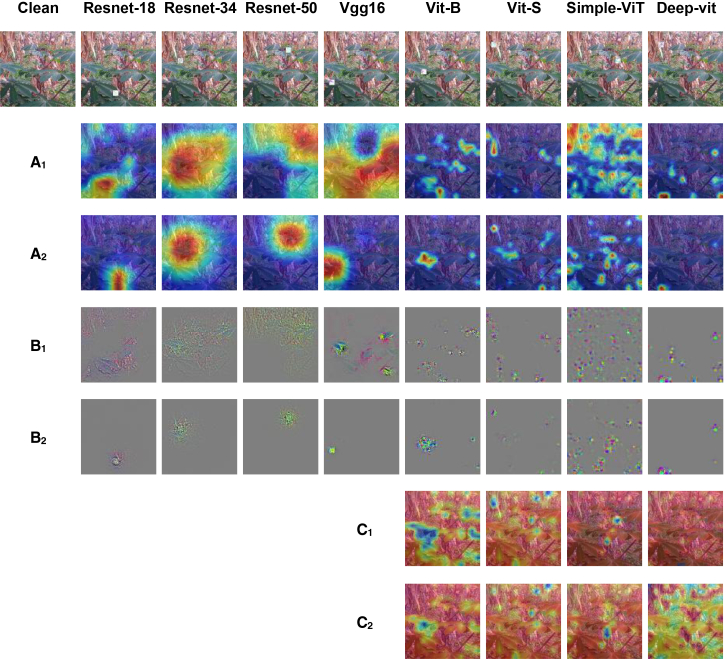
APA attack visualization on CNN and ViT models

**Figure 11:**
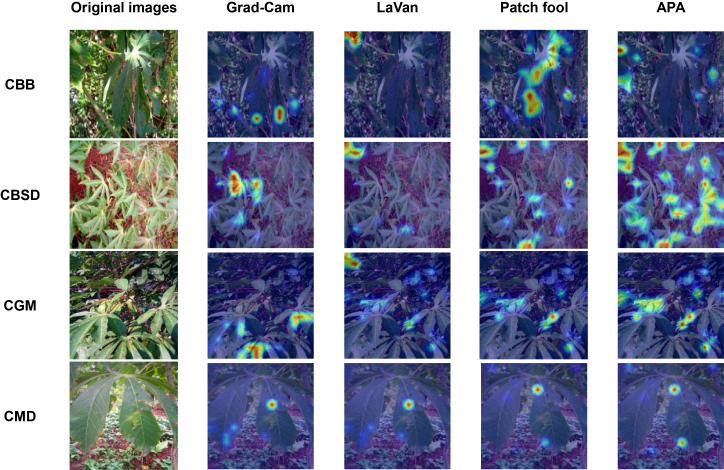
Feature visualization results of each patch attack

Figure 10 illustrates the results of the APA attack on various models. The clean image is correctly classified by all models, while the adversarial examples (AEs) successfully mislead the models. (1) A1 and A2: Grad-CAM visualizations for clean (A1) and adversarial (A2) samples, showing how the APA attack alters the models’ focus and attention. (2) B1 and B2: Feature map comparisons between clean (B1) and adversarial (B2) samples, generated from the first block of CNNs and attention blocks of ViTs, highlighting the disruption caused by the attack. (3) C1 and C2: Attention maps for clean (C1) and adversarial (C2) samples, derived from the final attention or convolutional layers, demonstrating the shift in attention caused by the APA attack. To evaluate the impact of different adversarial attack methods on the ViT-based cassava leaf disease classification model, we used Grad-CAM to visualize the output from the final attention module. We conducted visualizations for four disease types (CBB, CBSD, CGM, CMD) to assess the effectiveness of each attack in Figure 11. The red regions indicate the key areas where the model focuses during classification.

Comparing the three attack methods (LaVAN, patch-fool, and APA), it is observed that LaVAN and patch-fool attacks sometimes divert the model’s attention toward irrelevant background or healthy regions, having a weaker effect on the diseased areas.

In contrast, the APA method more effectively disrupts the model’s attention distribution, forcing the focus to shift to incorrect non-target areas, demonstrating a stronger attack effect. The visualizations indicate that under the APA method, the model’s attention on the diseased regions is more dispersed, showing that our approach is more disruptive and effective compared to LaVAN and patch-fool.

**Figure 12:**
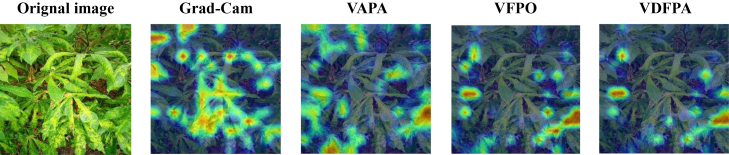
Visualization results of ablation experiments for the proposed methods

The visualization of the proposed framework in the ViT-B model is presented in Figure 12, where the highlighted regions represent the model’s attention distribution after applying adversarial attacks. In the Grad-CAM baseline, lesion areas are highlighted, but some attention is still directed toward the background. In the variance-aware patch attack (VAPA) section, the attack successfully shifts the model’s focus, causing prominent lesion areas to be overlooked and reducing background interference. The AFPO section, through color and texture modifications, further reduces the model’s focus on the lesion areas. The VDFPA section, combining both methods, shows the fewest highlighted regions, indicating the most efficient attack.

The results indicate that all three sections effectively redirect the model’s attention away from lesion areas. As adversarial perturbations increase, the number of highlighted regions decreases, reflecting improved attack efficiency. The variance-driven hybrid feature patch attack (VDFPA) section, in particular, most effectively minimizes the model’s focus on lesion areas, highlighting its effectiveness in enhancing adversarial attack performance.

**Figure 13:**
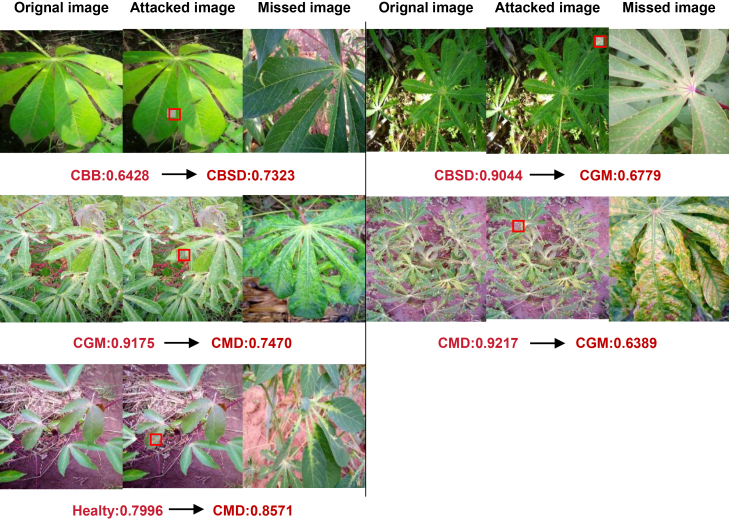
Misclassification results of cassava diseases after APA attacks

Figure 13 illustrates the classification results of cassava diseases before and after adversarial attacks. The original images are correctly classified, while the attacked images are misclassified into different disease categories. The results demonstrate that adversarial attacks effectively induce misclassification, highlighting the model’s vulnerability to perturbation.

## Discussion

**Figure 14:**
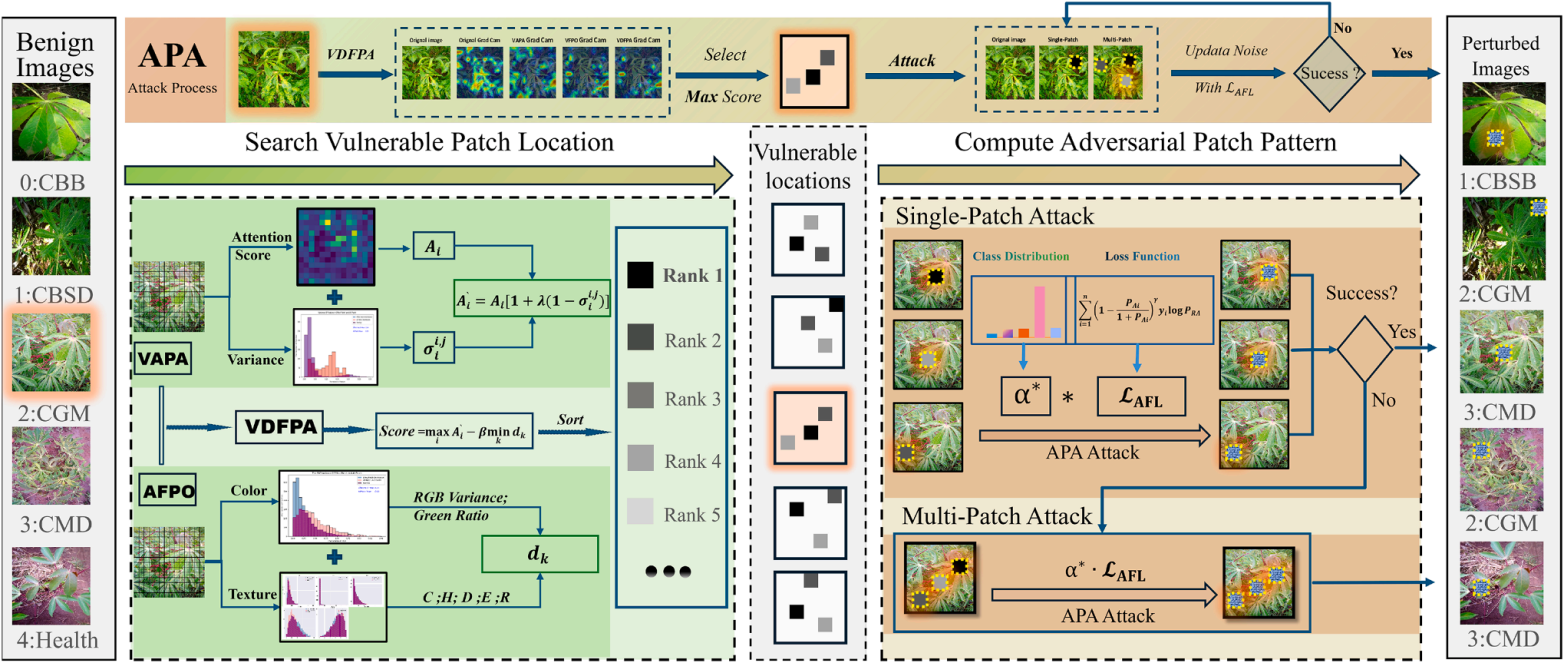
APA experimental flow chart

**Figure 15:**
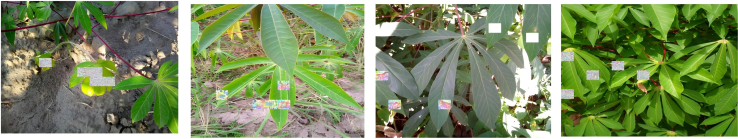
Overlay effect image of an effective patch

Figure 14 illustrates the overall APA framework, including vulnerable patch location search, adversarial patch pattern optimization, and the generation of perturbed images. Figure 15 shows representative perturbed images generated by APA, illustrating how adversarial patches are placed on cassava leaf images. The results indicate that patch location plays a key role in attacking plant disease recognition models. A close attention response does not always correspond to an effective patch location. Some highly attended regions lie on leaf boundaries or visually mixed areas. These regions may affect model prediction, but they do not always provide stable support for localized perturbations. Feature variance helps reduce this mismatch by favoring patches with more consistent internal structure. In this setting, lower variance corresponds to more coherent local features and leads to more stable patch placement.

This observation also helps explain why existing patch attacks may perform less effectively on plant disease images. Prior studies have shown that localized perturbations can strongly affect visual models,^72^^,^^73^ and that ViT models are sensitive to patch-level modifications because of their token-based structure.^26^^,^^29^ However, plant disease images often contain irregular leaf shapes, mixed visual patterns, and less clearly separated semantic regions. These properties have also been reported as key challenges in leaf-level analysis and broader agricultural vision tasks.^83^ Under these conditions, patch selection based only on model response may be insufficient. Additional visual constraints, such as feature consistency, can improve the reliability of patch localization.

Compared with representative patch attack methods such as LaVAN and patch-fool, APA does not aim to replace gradient- or attention-based optimization. Instead, it extends this line of work by introducing agricultural image priors into patch localization. LaVAN focuses on localized visible perturbations, while patch-fool exploits token-level attention vulnerability in ViT models. APA follows the same localized attack setting but further considers intra-patch feature coherence and simple plant-related visual cues. This distinction is important in plant disease images, where high model response alone may not correspond to stable or semantically meaningful patch locations.

The patch-level transformation analysis further examines the stability of generated patches under local appearance changes. When moderate variations in rotation, scale, and brightness are introduced, all methods show some degradation in attack effectiveness. However, APA maintains lower RA and shows smaller performance variation across these settings. This suggests that the proposed patch selection strategy is less sensitive to mild local appearance perturbations.

Overall, the findings suggest that adversarial robustness in plant disease recognition should be analyzed not only from the model side, but also from the structure of agricultural images. Patch-level vulnerability is shaped by attention response, local feature consistency, and category-dependent visual patterns. This provides a useful basis for future robustness-oriented model design.

### Limitations of the study

Despite these observations, the current study remains a controlled digital robustness evaluation. The patch-level transformations simulate mild local appearance changes, but they do not fully cover physical deployment factors such as printing quality, camera distance, viewing angle, leaf motion, illumination shifts, occlusion, and environmental noise. In addition, APA uses model-side information for patch placement, which is suitable for worst-case robustness diagnosis but does not represent a complete black-box or physical world attack pipeline. Future work should evaluate printed or camera-captured patches, category-level universal patches, cross-dataset transferability, and defense strategies against localized perturbations in field-oriented agricultural imaging systems.

## Resource availability

### Lead contact

Requests for further information and resources should be directed to and will be fulfilled by the lead contact, Youtao Zhang (youtao@pitt.edu).

### Materials availability

This study did not generate new unique reagents.

### Data and code availability

• The cassava leaf disease dataset used in this study is publicly available from the Kaggle Cassava Leaf Disease Classification competition [Dataset URL: L: https://www.kaggle.com/c/cassava-leaf-disease-classification/data (accessed on 1 June 2022)].
• The source code, configuration files, attack scripts, and evaluation scripts for reproducing the main results have been deposited at https://doi.org/10.5281/zenodo.21202840. The DOI/accession number is provided in the key resources table.
• Any additional information required to reproduce the results is available from the lead contact upon reasonable request.

## Acknowledgments

This work was supported by the 10.13039/501100001809National Natural Science Foundation of China (grant nos. 32472007, 62306008, and 62301006), the 10.13039/501100012166National Key Research and Development Program of China (grant no. 2023YFD1802200), the Key Project of Anhui Province’s Science and Technology Innovation Tackle Plan (grant no. 202423k09020040), the Natural Science Research Project of the Education Department of Anhui Province, China (grant no. 2023AH051020), the 10.13039/501100003995Natural Science Foundation of Anhui Province (grant no. 2308085MF21), the University Synergy Innovation Program of Anhui Province (grant no. GXXT-2022-046), the Provincial Quality Engineering Project for the “Four New” Research and Reform Practice in Higher Education of Anhui Province (grant no. 2023sx022), and the Philosophy and Social Science Research Project of Anhui Higher Education Institutions (grant no. 2023AH050966).

## Author contributions

Conceptualization: Y.W., L.G., and Y.Z.; methodology, Y.W. and M.W.; software, Y.W., M.W., and Z.Z.; data curation, M.W., W.Y., and Z.Z.; validation, X.J., Y.D., L.L., Y.Y., W.Y., and Z.Z.; formal analysis, Y.W.; visualization, Y.W., X.J., and Y.D.; writing—original draft, Y.W.; writing—review and editing, Y.Z.; funding acquisition, L.G. and Y.Y.; project administration, L.G.; supervision, Y.Z.

## Declaration of interests

The authors declare no competing interests.

## Declaration of generative AI and AI-assisted technologies in the writing process

During the revision of this work, the author(s) used ChatGPT to improve the readability of some sections of the manuscript. Following the use of this tool, the author(s) carefully reviewed, edited, and approved the final content. The author(s) take full responsibility for the content, accuracy, and integrity of the published work.

## STAR★Methods

### Key resources table

**Table:** 

REAGENT or RESOURCE	SOURCE	IDENTIFIER
**Deposited data**		

Cassava Leaf Disease Dataset	Kaggle	Dataset URL: L: https://www.kaggle.com/c/cassava-leaf-disease-classification/ data

**Software and algorithms**		

Research Code	GitHub	https://doi.org/10.5281/zenodo.21202840

### Experimental model and study participant details

This study did not involve human participants, animals, cell lines, microbial strains, or primary cell cultures. The experimental data consisted of publicly available RGB images from the Cassava Leaf Disease Classification dataset. The image subjects were cassava leaves (Manihot esculenta) collected under field conditions and annotated into five categories: CBB, CBSD, CGM, CMD, and healthy leaves.

The dataset contains 21,397 annotated images and was used only for computational model training, adversarial patch generation, and robustness evaluation. No new plant samples were collected, and no intervention was performed on living plants. Information related to age, sex, gender, ancestry, race, and ethnicity is not applicable. Institutional approval was not required because this study used a publicly available image dataset and did not involve human or animal subjects.

### Method details

To address the above challenges,our method is designed according to three considerations. First, patch selection should not rely only on raw attention scores. Model sensitive regions are not always stable locations for local perturbations. Second, intra patch feature variance can provide a useful cue for local visual coherence. Patches with lower variance often correspond to more consistent plant regions, which may support more stable perturbation placement. Third, class imbalance can affect attack difficulty across disease categories. A dominant class may show different vulnerability from underrepresented disease classes, making a class aware attack objective necessary.

Based on these considerations, we propose APA, a tailored adversarial patch framework for agricultural disease recognition. An overview of the proposed framework is shown in Figure 14. APA follows an untargeted attack paradigm. It aims to reduce overall classification accuracy without specifying a particular misclassification target. In this paper, the term “target” refers to the strategic selection of patch positions or optimization mechanisms, rather than traditional targeted classification attacks. In this section, we introduce APA, an adversarial patch framework tailored for ViT-based plant disease models. APA includes two key modules:(1) Variance-Driven Feature Patch Attack (VDFPA), enhances patch placement by combining attention scores with local feature variance and agricultural texture cues;(2) Adversarial Focal Loss (AFL), applies adaptive weighting during optimization to focus on robust classes and address class imbalance. These modules jointly improve attack effectiveness under the complex visual and categorical conditions of agricultural disease detection.

ViT models are typically robust to random local noise. However, their self-attention structure can be highly sensitive to adversarial perturbations on a single patch. This observation motivates our investigation into how localized changes influence the model’s global reasoning.This idea is inspired by word substitution attacks^84^^,^^85^ which attack by replacing words with synonyms in the transformer model in NLP tasks. Similarly, image patching plays a similar role in ViT.

In the field of agricultural disease identification, diseases are often concentrated in specific areas of the plant. By applying customized adversarial perturbations to image patches in these specific regions, the model’s recognition ability can be more effectively disrupted, increasing the success rate of the adversarial attack. This approach not only reveals the vulnerability of the ViT model in agricultural disease detection, but also provides an important reference for designing a more robust agricultural disease detection system.

#### Problem formulation

In general, the attacks in our work focus on the context of image classification. We use $X\in R^{H\times W\times 3}$ to denote the distribution of images, where each image *x* ∈ *X* has width *W*, height *H*, and number of channels 3 (RGB three-channels). The label space, denoted by *Y* = {0, 1, …, *N* − 1}, is defined as the set of all possible class labels, with *N* being the number of classes. The model, *f*:*X* → *Y*, takes an image as input and predicts the class label *y* ∈ *Y* that maximizes the objective as Equation 1:

(Equation 1) $$\begin{array}{c} \arg\max_{p}L\left(f\left(F\left(x,p,L\right)\right),Y\right) \end{array}$$

With L specifying the location of the adversarial patch *p* within the larger image *x*, F a function to apply the patch onto the image (i.e., overlays the patch *p* onto the image *x* at a specified location *L*), and *f* being the target model.

#### Variance-aware patch attack (VAPA)

We design a new feature, the variance-based attention score improvement module, called Variance-Aware Patch Attack (VAPA). We tested several Vision Transformer models, including ViT and DeiT, which used pre-trained weights from the ImageNet dataset and fine-tuned the finished pre-trained models in subsequent experiments. The experiments were evaluated on patches extracted from the Cassava dataset.

#### Gradient attacks under dot-product attention

Gradient based attack methods, such as PGD, FSGM, and BIM, generate adversarial examples by computing the gradient of the loss function (e.g., cross-entropy) with respect to the input data. These examples aim to mislead the model into making incorrect predictions. By identifying patches with large gradient values, attackers can target areas that significantly impact model loss, directly influencing the model’s output. However, most gradient based methods are designed for CNNs. When applied to ViTs, the self-attention mechanism introduces additional complexity in how gradients affect the model.

In ViTs, the value vector *V* is computed as $V=XW_{V}^{h}$, where *X* is the input, and $W_{V}^{h}$ is the weight matrix for attention head *h*. Gradients for the self-attention mechanism, such as ∇_*x*_SelfA^*h*^(*X*), require backpropagation through attention layers. These gradients involve two components: (∇_*X*_*A*^*h*^(*X*))*X* and *A*^*h*^(*X*)1_*x*_, where *A*^*h*^(*X*) represents attention weights. Studies (e.g., Lovisotto et al.^29^) show that in ViTs, the gradient component (∇_*X*_*A*^*h*^(*X*))*X* is often 20 times smaller than *A*^*h*^(*X*)1_*x*_. As a result, attention weights *A*^*h*^(*X*) can be approximated as constants during gradient calculations.

Traditional gradient based attacks such as PGD, FGSM, and BIM primarily manipulate the value vectors, focusing on linear perturbations. However, these methods typically overlook the nonlinear interactions mediated by attention weights in ViT architectures. This limitation motivates the development of attack strategies that explicitly incorporate attention-driven vulnerabilities.

To address these limitations, this study enhances adversarial attack strategies by leveraging attention scores and incorporating feature vector variance. This approach captures the nonlinear effects of attention propagation while considering feature variability across patches in agricultural disease images, thereby improving the attack’s effectiveness against ViT models.

#### ViT-attention mechanism

Given an input image $X\in R^{H\times W\times 3}$ with resolution *H*×*W* and three channels, ViT^10^ reshapes *X* into a sequence of patches of size *P*×*P*, flattening them into vectors. The input image can thus be represented as $x^{\left(0\right)}\in R^{P\times P\times N}$, where $N=\frac{H\times W\times C}{P^{2}}$ is the number of patches.

ViT computes attention using the scaled dot-product mechanism.^20^ Attention weights are calculated as the dot product between the queries (*Q*) and keys (*K*), followed by a softmax operation over the key dimension, and then multiplied by the values (*V*).

(Equation 2) $$\begin{array}{c} \text{Attention}\left(Q,K,V\right)=\text{softmax}\left(QK^{T}\right)V \end{array}$$

Here, $Q,K,V\in R^{n\times d_{\text{model}}}$ represent matrices of *n* queries, keys, and values, respectively. As described by Vaswani et al.,^20^ when *d*_model_ is large, the dot-product magnitudes between queries and keys can be significant, causing the softmax function to enter a saturated regime with small gradients. To mitigate this, scaled dot-product attention is applied during training:

(Equation 3) $$\begin{array}{c} \text{Scaled}\;\text{Attention}\left(Q,K,V\right)=\text{softmax}\left(\frac{QK^{T}}{\sqrt{d_{k}}}\right)V \end{array}$$

In practical implementations, employing more than one attention head by linearly projecting the queries, keys, and values multiple times to dimensions *d*_*k*_, *d*_*k*_, and *d*_*v*_ has proven advantageous. The output of the *h*-th attention header is:

(Equation 4) $$\begin{array}{c} {\text{head}}_{h}=\text{Attention}\left(QW_{Q}^{h},KW_{K}^{h},VW_{V}^{h}\right) \end{array}$$

The output from the *h*-th attention head is given by:

(Equation 5) $$\begin{array}{c} A_{H}^{h}\left(Q,K,V\right)=\text{softmax}\left(\frac{QW_{Q}^{h}{\left(KW_{K}^{h}\right)}^{T}}{\sqrt{d_{k}}}\right)VW_{V}^{h} \end{array}$$

where $W_{Q}^{h},W_{K}^{h},W_{V}^{h}\in R^{d_{\text{model}}\times d_{k}}$ are the learned projection matrices. The outputs from each attention head are concatenated and subsequently multiplied by another learned projection matrix $W_{o}\in R^{Hd_{v}\times d_{\text{model}}}$, to generate the final output.

(Equation 6) $$\begin{array}{c} \text{MultiHead}\left(Q,K,V\right)=\text{Concat}\left({\text{head}}_{1},\ldots ,{\text{head}}_{h}\right)W_{o} \end{array}$$

The attention score scores = *QK*^*T*^ measures the similarity between queries and keys, and attention weights, computed via softmax, indicate the importance of each key to a query:

(Equation 7) $$\begin{array}{c} {\text{AttentionWeight}}_{ij}=\text{softmax}\left(\frac{Q_{i}K_{j}^{T}}{\sqrt{d_{k}}}\right) \end{array}$$

The final attention output vector is the weighted sum of the values *V*, combining query and key importance:

(Equation 8) $$\begin{array}{c} \text{output}=\text{weights}\cdot V \end{array}$$

This mechanism enables the model to dynamically focus on different parts of the input, capturing complex relationships and improving sequence data processing.

#### Determine x via variance-aware patch selection

In the ViT model, the first layer embeds the input patches into representation vectors with higher dimensions, which are denoted as *x*^(1)^. To improve performance, ViT models typically introduce additional representation vectors, such as category vectors or distillation vectors. A typical ViT model contains *L* layers, where each layer has the same dimension. We denote the representation vector matrix of the first layer as $x^{\left(l\right)}\in R^{n\times d}$, where *l* ∈ {1, 2, …, *L*}, *n* = *N* + 1 denotes the number of vectors, *d*_model_ is the length of each vector, and *l* denotes the number of the layer.

Define $a^{\left(l,h,i\right)}=\left[a_{1}^{\left(l,h,i\right)},\cdots ,a_{n}^{\left(l,h,i\right)}\right]\in R^{n}$ as the attention distribution for the *i*-th element in the *h*-th head of the *l*-th layer. For any layer *l*, let:

(Equation 9) $$\begin{array}{c} s_{j}^{\left(l\right)}=\sum_{h,i}a_{j}^{\left(l,h,i\right)} \end{array}$$

Here, $s_{j}^{\left(l\right)}$ quantifies the significance of the *j*-th element in the *l*-th layer by summing its self-attention contributions to other elements. This value highlights its overall importance in the layer. To better deceive ViT, we choose the most influential patch *x* in the last layer of self-attention (i.e., layer 12), which is determined by $\arg\;\max_{j}s_{j}^{\left(l\right)}$. The reason for choosing layer 12 is that as the number of layers increases, the model mixes the information in the self-attention mechanism more appropriately, and the last layer is able to synthesize the global information and identify more representative and important patches, making the adversarial perturbations more effective, as described in the hyperparameter analysis section.

#### Patch selection via variance-aware attention scoring

In the ViT model, the attention score-based attack method is one of the common adversarial attack strategies, which mainly relies on the attention score to evaluate the importance of each patch. The image is divided into several equal-sized patches, each of which is converted into a vector input for the model. In this way, the feature representation of each position is not limited to the information of the surrounding area but also contains the global information of the entire image. Therefore, the feature is particularly important in ViT. Feature vectors at different positions must capture varied aspects of the image. This diversity ensures that the model can interpret the image accurately. As a result, the model can perform tasks such as classification or other objectives effectively. However, the original attack method based on attention score only considers the correlation between patches and ignores the characteristics of the internal features of patches. In some cases, it may ignore patches with low feature diversity but great impact on the prediction results of the model. In addition, relying solely on the attention score to Adversarial attacks may be susceptible to interference and not be robust enough. To address this limitation, we introduce feature variance as a complementary descriptor.

The feature variance can indicate the degree of dispersion of the eigenvector value from the mean value. In the VIT model, the input image is segmented into fixed-size patches; each patch is flattened into a one-dimensional vector and mapped to a fixed-dimensional feature vector by a linear transformation. Patches at different locations may contain different content (e.g., one patch may be a leaf, while another may be soil), and these will be represented differently in the feature space. Some patches may contain more textured and detailed information, while others may be relatively smooth or simple.

We conducted a series of preliminary experiments to explore the relationship between feature vector variance and attack effectiveness. In general, patches with higher attention scores tend to contain more focused feature information, and their feature vector variance may be smaller. Experimental results confirm our suspicions, as described in the experimental design verification section.

We compute the variance of feature vectors from the self-attention model’s final layer, Layer 12, to characterize each patch. By integrating feature variance into the attention score calculation, we aim to reweight patch importance, prioritizing those with high attention and semantically consistent content. Specifically, we compute the adjusted attention score as show in Eq. 10.

##### Adjusted attention scores

(Equation 10) $$\begin{array}{c} A_{i}^{\prime }=A_{i}\left(1+\lambda \left(1-\sigma_{i,j}^{2}\right)\right) \end{array}$$

Where *A*_*i*_ is the original attention score of patch *i*, $\sigma_{i,j}^{2}$ is the normalized variance of its feature vector, and *λ* is a hyperparameter that controls the strength of variance modulation. In our experiments, the normalized variance values are consistently smaller than 1, as shown in Figures 5 and 6. Thus, $\left(1-\sigma_{i,j}^{2}\right)$ remains non negative in the present setting and can be interpreted as a feature coherence factor.

A lower feature variance indicates more consistent intra patch features and clearer local structure. The variance term increases the adjusted attention score of coherent patches and suppresses visually heterogeneous regions. As a result, the strategy prioritizes patches that are both highly attended by the model and locally coherent in feature space. Its behavior under different feature scales or image domains remains to be further studied.

##### Original attention score

(Equation 11) $$\begin{array}{c} A_{i}=\sum_{h=1}^{\text{num}\_\text{heads}}\frac{Q_{i}^{h}{\left(K_{i}^{h}\right)}^{T}}{\sqrt{d_{k}}} \end{array}$$

Where $Q_{i}^{h}$ and $K_{i}^{h}$ are the query and key representations of the *i*-th patch at the *h*-th attentional head, respectively, and *d*_*k*_ is the scaling factor;

##### Feature mean and feature variance

To operationalize this, we calculate feature vectors *T*_*i*,*j*,*k*_ from the final attention layer (Layer 12) as:

(Equation 12) $$\begin{array}{c} \mu_{i,j}=\frac{1}{D}\sum_{k=1}^{D}T_{i,j,k} \end{array}$$

(Equation 13) $$\begin{array}{c} \sigma_{i,j}^{2}=\frac{1}{D-1}\sum_{k=1}^{D}{\left(T_{i,j,k}-\mu_{i,j}\right)}^{2} \end{array}$$

where *μ*_*i*,*j*_ is the mean of the *j*-th patch of the *i*-th sample, *D* is the embedding dimension (e.g., 768), and *T*_*i*,*j*,*k*_ is the value for the *k*-th dimension of patch *j* in sample *i*.

##### Select key patches to attack

Finally, we select the patch with the highest $A_{i}^{\prime }$ values for adversarial perturbation:

(Equation 14) $$\begin{array}{c} \left(j^{\prime },k^{\prime }\right)=\arg\max_{i}A_{i}^{\prime } \end{array}$$

Eq. 14 selects top-scoring patch based on the improved attention score $A_{i}^{\prime }$. This selection aims to identify patch that are both semantically informative and structurally stable. This adjustment provides a more robust and semantically guided way to identify vulnerable patches, especially in visually heterogeneous agricultural scenes.

#### Agri-Feature Patch Optimization (AFPO)

Existing adversarial attack optimization algorithms, such as gradient based and attention-score-based, mainly rely on the internal mechanism of the model, focusing on the parameter sensitivity and gradient information of the model without fully combining with the characteristics of the data itself to further explore and analyze. Agricultural disease data have unique features; for example, the images mainly contain green leaves with significant color features. Therefore, we try to find relevant features through color metrics (e.g., RGB variance and green percentage). However, in practice, diseases are often small, and images of leaf diseases exhibit distinct local features in texture and space with minimal impact on the output of color (RGB) features. Relying solely on color features may not accurately extract relevant information. To solve this problem, we propose a hybrid feature extraction method that combines color and texture features. Through preliminary experimental analysis, we integrate color features (RGB variance, proportion of green) and texture features (contrast, dissimilarity, homogeneity, energy, and correlation) to establish criteria for an effective feature set. Additionally, we utilize similarity indices to optimize the search for patch positions. The method can better capture the complex characteristics of agricultural disease images, optimize the search strategy for effective patches, and improve the effectiveness of patch attacks. This data-driven search method, which integrates the unique characteristics of agricultural disease data, provides a more precise and effective pathway for adversarial attacks.

Since an exhaustive search method is both computationally intensive and time-consuming, we randomly sampled 1,000 images in the training set to form a subset, traversed it, extracted the relevant features of effective patches, constructed a feature vector, and finally used the similarity evaluation criteria to narrow the search space, thereby significantly improving attack efficiency. By conducting exhaustive attacks on 1,000 cassava images, we identified patches that successfully compromised the system. Specifically, we iterated through all possible patch locations (*p*_*i*,*j*_) of each image, applied an adversarial perturbation (*δ*_*i*,*j*_) to each location, and evaluated whether the perturbed image (*X* + *δ*_*i*,*j*_) can successfully fool the target model (*f*).

The conditions for a successful attack are as follows:

(Equation 15) $$\begin{array}{c} f\left(x+\delta_{i,j}\right)\neq f\left(x\right) \end{array}$$

Based on the valid patches, we further extract their seven key features, including color features: RGB variance, green ratio, and texture features: contrast, dissimilarity, homogeneity, energy, and relevance. These features can be represented by a set of vectors in Eq. 16:

(Equation 16) $$\begin{array}{c} T=\left[V_{RGB},G_{R},C,D,H,E,R\right] \end{array}$$

#### Color feature extraction

By analyzing the valid patches, we found that most of the valid patches are concentrated in the leaf area of the original images (Figure 15). To quantify color uniformity, we calculate the RGB variance by first computing the variance of each color channel (R, G, B) within a patch and then averaging them. This average variance serves as a holistic indicator of color stability across channels. We chose the average rather than the minimum or maximum value to avoid bias toward isolated fluctuations in a single channel, which may be caused by noise or illumination changes. It showed that the average variance more reliably highlights uniformly colored regions, particularly in green plant areas such as leaves. This metric therefore provides a practical basis for selecting visually consistent and semantically meaningful patch regions. Based on this observation, we can draw the following conclusions.

(1) RGB Variance: The RGB variance measures the color consistency within each patch of an image. We calculate the variance for each of the red, green, and blue channels and average them. The red channel variance is calculated as the average of the squared differences between each red value *R*_*i*_ in the patch and the mean red value $\bar{R}$. We calculate the variance for green and blue channels similarly.

The overall RGB variance *V*_RGB_ is the average of the variances for the red, green, and blue channels, representing the color stability of the patch. A lower RGB variance generally indicates a more uniform color distribution, a characteristic typically found in areas with consistent colors (e.g., within leaf regions) rather than at boundaries (e.g., the junctions between leaves and stems or between leaves and soil).

(2) Green ratio: The green ratio represents the proportion of green pixels in the patch. It measures the ratio of the number of green pixels in the patch to the total number of pixels. A high green ratio generally indicates that the patch consists mainly of green areas, such as plant leaf parts. The green ratio is particularly important in agricultural image analysis because it can help identify and distinguish plant leaves from non-leaf areas, providing valuable information for further image processing and analysis by quantifying the degree of green cover. Therefore, we choose RGB variance and green percentage as the color features to be extracted. It is calculated as follows:

(Equation 17) $$\begin{array}{c} G_{R}=\frac{\sum_{i=1}^{N}1\left(G_{i}>R_{i}\;\text{and}\;G_{i}>B_{i}\right)}{N} \end{array}$$

#### Texture feature extraction

Color analysis often relies on the color distribution within an image; however, this feature is susceptible to inconsistencies caused by lighting variations, movement, and camera shake, among other factors. To enhance robustness in agricultural disease analysis, texture features are introduced, capturing statistical or structural pixel relationships that convey detailed image attributes. Texture analysis approaches include statistical, structural, model-based, and transformation-based methods.^86^^,^^87^ Among these, the Gray-Level Co-occurrence Matrix (GLCM), a statistical method, is widely used for texture extraction, as it captures spatial dependencies of textures within the image.

In analyzing agricultural disease images, GLCM extracts second-order statistical measures with low computational complexity, rotational invariance, and multi-scale feature stability. Haralick et al.^86^ defined 14 texture features; this study selects five key indicators, contrast (C); homogeneity (H); dissimilarity (D); energy (E) and correlation (R), which effectively reflect texture characteristics of agricultural disease images and facilitate the identification of significant patch locations.

#### Similarity assessment of patch features

To identify patches that best match the characteristics of previously validated effective patches, we compute a similarity score based on seven features: RGB variance (*V*_RGB_), green ratio (*G*_*R*_), and five texture features (contrast *C*, dissimilarity *D*, homogeneity *H*, energy *E*, and correlation *R*). These are used to construct a feature vector *X*_*i*_ for each patch *i*:

(Equation 18) $$\begin{array}{c} X_{i}=\left[V_{\text{RGB}},G_{R},C,D,H,E,R\right] \end{array}$$

We then define a target feature vector:

(Equation 19) $$\begin{array}{c} T=\left[V_{\text{RGB}}^{*},G_{R}^{*},C^{*},D^{*},H^{*},E^{*},R^{*}\right] \end{array}$$

extracted from previously successful patches, and calculate the Euclidean distance between *X*_*i*_ and *T* in Eq. 20:

(Equation 20) $$\begin{array}{c} d_{i}=\sqrt{\sum_{j=1}^{n}{\left(x_{ij}-T_{j}\right)}^{2}} \end{array}$$

Here, *d*_*i*_ denotes how similar patch *i* is to the target profile. The patch with the smallest *d*_*i*_ is considered the most similar, and the corresponding patch *i* is the optimal position, that is, the patch where the feature vector *X*_*i*_ is closest to the target feature vector *T*:

(Equation 21) $$\begin{array}{c} i_{\text{opt}}=\arg\min_{i}d_{i} \end{array}$$

The validity of the selected patch (*i*_opt_) in Eq. 21 is verified through attack experiments, and the set of target features is recorded and updated. The experimental results are shown in the experimental design verification section.

#### Variance-driven hybrid feature patch attack (VDFPA)

To further refine patch selection, we introduce a unified scoring function, referred to as the VDFPA approach. That integrates both the modified attention score $A_{i}^{\prime }$ (from VAPA) and the similarity distance *d*_*i*_ (from AFPO), where *i* indexes over all patch positions. Specifically, $A_{i}^{\prime }$ refines the attention score by incorporating variance constraints, aiming to maximize the modified score to reflect the model’s attention concentration on the target region. Conversely, *d*_*i*_, uses Euclidean distance to measure the color and texture differences in the target area, where a smaller value indicates better alignment with the reference target.

The final score for each patch in Eq. 22, which characterizes the VDFPA strategy, is defined as follows

(Equation 22) $$\begin{array}{c} {\text{Score}}_{i}=A_{i}^{\prime }-\beta d_{i} \end{array}$$

Where: $A_{i}^{\prime }$ represents the modified attention score, optimized through variance constraints to assess the model’s focus on the target region; *d*_*i*_ denotes the Euclidean distance for color and texture features, measuring the deviation between the target region and the reference. *β* are the weighting coefficients that balance the contributions of the attention score and the Euclidean distance.

The selected patch is:

(Equation 23) $$\begin{array}{c} i^{*}=\arg\max_{i}{\text{Score}}_{i} \end{array}$$

This unified strategy ensures that the chosen patch is both highly attended by the ViT model and semantically similar to historically effective patches in color-texture space. This design enhances the model’s performance in complex conditions, maintaining a balance between attention stability and feature matching accuracy.

#### Loss function under class imbalance

##### Adversarial attacks based on category imbalance

Category imbalance is a notable challenge in agriculture, especially when faced with disease datasets.^88^^,^^89^ Specifically, some disease categories had far more samples than others. Taking a typical crop disease detection dataset as an example, common leaf spot diseases may have tens of thousands of image samples, while rare diseases, such as root rot, may only have a few hundred samples. The cassava disease dataset used in our study also showed a similar imbalance problem, As shown in Figure 2; among them, the fourth disease category (CMD disease) accounts for 61.5% of the total dataset, while the proportions of the other disease categories are around 10%. Furthermore, we applied the cross-entropy loss function (which is widely used in the field of adversarial attacks, to evaluate the effectiveness of its attack on the cassava disease dataset. As shown in Section, Table 11, the attack success rate of CMD diseases, which account for the largest proportion, is only 13%. This finding suggests that the class imbalance problem in training data has become a key obstacle limiting the improvement of attack success rate. To solve this problem, we propose a new loss function, the Adversarial Focus Loss (AFL), based on the traditional cross-entropy loss. This feature is designed to increase the model’s focus on minority classes, thereby improving the overall effectiveness of the attack. It is an effective response to the challenges of class imbalance in real-world applications.

In the training process of classification models, gradient descent is usually used to minimize the loss function. Traditional loss functions, such as cross-entropy loss, tend to average over all samples when performing parameter updates, resulting in less focus on minority class samples in training. In a class-imbalanced dataset, samples from the majority class occupy the vast majority of the training data, which means that model parameter updates are more frequently influenced by samples from the majority class in each training cycle (epoch). This advantage of update frequency allows the model to learn the features of a few classes more sufficiently, improving the classification accuracy for these classes.

In the context of adversarial attack training, since the majority of class samples cover a larger portion of the feature space, the model is able to learn and generalize these features more efficiently, thus clearly and robustly defining its decision boundaries. In feature space, due to the clear and stable decision boundaries of most class samples, these boundaries are not easily affected by minor perturbations. Thus, for most classes of samples, adversarial attacks usually require larger perturbations to induce the model to misclassify, thus increasing the difficulty of the attack. This phenomenon highlights the impact of class imbalance on model generalization and adversarial attack effectiveness and underscores the importance of balancing class distribution in future adversarial training strategies.

Class imbalance is typically defined as a significant difference in sample size between different classes in a dataset. If this imbalance is not handled properly, training classifiers on such datasets often results in classifiers biased in favor of the majority class, thereby reducing the model’s prediction performance for the minority class. To correct this bias effect, scholars usually use data level and algorithm level methods^90^ to correct this bias effect. Data-level methods refer to the resampling of data.^91^ However, this method usually changes the data distribution or runs the risk of overfitting. Also, resampling can be accomplished using classifier integration, such as iterative sampling,^92^ Generating Adversarial Networks^93^ Methods. In comparison, algorithm-level methods are usually more intuitive and can introduce weights for each category or sample, emphasizing the minority class in the target, thereby guiding the algorithm behavior. A common approach is to define the weight parameters as hyperparameters of the category,^29^ but it is often difficult to obtain optimal heuristic values.

Currently, some researchers have begun to explore how the two factors, category imbalance and difficult samples, jointly affect the loss function. In the application of Focal Loss (FL), the inverse function of sample output probability is used to adjust the weight of each sample in the loss, especially to assign higher weights to difficult samples, in order to promote the network to learn these samples more effectively. However, these studies are mainly from the perspective of improving classification accuracy, and for the adversarial attack area, no studies have been conducted to optimize attack efficiency from the perspective of category imbalance. Therefore, in this paper, we draw on the idea of difficult example mining, and for the samples that are difficult to attack, we design the adversarial focus loss function, which is used for the task of adversarial attack in the agricultural disease recognition model. In contrast to traditional cross-entropy loss functions that allow for average gradient updating, the adversarial focus loss function facilitates perturbation updating by enabling dynamically scaled gradient updating, thus providing optimization in dealing with class imbalances and difficult samples. This approach improves the success rate of adversarial attacks and, to the best of our knowledge, is the first exploration and experiment of class imbalance in adversarial attacks.

#### Adversarial focus loss

In this study, we propose an Adversarial Focal Loss (AFL) to address the challenges posed by adversarial attacks, class imbalance, and varying difficulties in attacking certain categories.

##### Weighted cross entropy loss

To handle class imbalance, a common method adds a weighting factor *α*_*i*_. This balances the loss between samples in categories with different quantities. This method is called Weighted Cross Entropy (WCE), as shown in:

(Equation 24) $$\begin{array}{c} L_{\text{WCE}\left(p,y_{i}\right)}=-\sum_{i=1}^{N}\alpha_{i}y_{i}\;\log\left(p_{i}\right) \end{array}$$

The weight *α*_*i*_ represents the weight for category *i*, where *α* can be treated as a constant or the reciprocal of category frequency *f*. For clarity, we refer to this approach as Adaptive Weighted Cross Entropy (WCE*α*). The WCE*α* can be formulated as follows:

(Equation 25) $$\begin{array}{c} L_{{\text{WCE}}_{\alpha }\left(p,y_{i}\right)}=-\sum_{i=1}^{N}\frac{1}{f_{i}}y_{i}\;\log\left(p_{i}\right) \end{array}$$

Here, *f*_*i*_ is the sample frequency of category *i*. To avoid extreme category unbalance, where the weight adjustment between categories is highly variable, we further normalize *α*_*i*_, to ensure that the sum of *α* values of all categories is 1. Therefore, *α*_*i*_ is adjusted to:

(Equation 26) $$\begin{array}{c} \alpha_{i}^{*}=\frac{\frac{1}{f_{i}}}{\sum_{j=1}^{N}\frac{1}{f_{j}}} \end{array}$$

WCE*α*∗ can be formulated as follows:

(Equation 27) $$\begin{array}{c} L_{{\text{WCE}}_{\alpha^{*}}\left(p,y_{i}\right)}=-\sum_{i=1}^{N}\left(\frac{\frac{1}{f_{i}}}{\sum_{j=1}^{N}\frac{1}{f_{j}}}\right)y_{i}\;\log\left(p_{i}\right) \end{array}$$

By assigning higher weights to pixels in smaller classes, the method emphasizes their importance over larger classes. However, for adversarial attacks in agricultural diseases, this up-weighting strategy for smaller classes does not lead to better model optimization, as shown in our experiments in the hyperparameter analysis section.

##### Adversarial focal loss (AFL)

Yao et al.^23^ propose FL, which reduces the loss for well-classified examples. This shifts the focus to harder negatives, as shown in Eq. 28. To achieve this, the factor ${\left(1-p_{t}\right)}^{\gamma }$ is added to the cross-entropy loss. Here, *γ* is a focusing parameter (*γ* ≥ 0) that controls the level of focus.

(Equation 28) $$\begin{array}{c} L_{\text{FL}}\left(p,y_{i}\right)=-\sum_{i=1}^{N}{\left(1-p_{t}\right)}^{\gamma }y_{i}\;\log\left(p_{i}\right) \end{array}$$

The value *p* ∈ [0,1] represents the model’s predicted probability for each class, and *p*_*t*_ is the probability of the correct class.

For the situation of the adversarial attack domain, we further replace *p*_*i*_ by *p*_Ai_, where *p*_Ai_ is the probability of the model output after an attack on category *i*. A higher *p*_Ai_ means a higher probability that the attack is unsuccessful (see the experimental setup section for more details). This allows us to focus the attack model’s attention more on categories that are difficult to attack, i.e., to give more of the adversarial loss to samples that are hard to attack. When *γ* = 0, a focal loss (AFL) is equivalent to cross-entropy (CE) loss, increasing *γ* enhances the modulating factor’s effect, with *γ* = 1 yielding optimal results in our experiments.

Additionally, an *α*∗-balanced version of FL, referred to as AFL in this paper, is introduced to give more weight to the smaller class, as shown in:

(Equation 29) $$\begin{array}{c} L_{\text{AFL}\left(p_{\text{Ai}},y_{i}\right)}=-\sum_{i=1}^{N}\alpha_{i}^{*}{\left(1-\frac{P_{Ai}}{1+P_{Ai}}\right)}^{\gamma }y_{i}\;\log\left(p_{\text{Ai}}\right) \end{array}$$

where $\alpha_{i}^{*}$ is defined by Eq. 26.

This approach has shown good results in multi-category adversarial attack tasks. Also, it can better deal with the problems of category imbalance and difficulty in attacking specific categories.

In the dataset used for this study, the number of CMD categories is approximately 7–10 times the average of the other categories. However, the actual appropriate magnification should be less than this average. This is because most hard examples tend to come from small categories, and increasing the weight parameter based only on category frequencies would result in over-amplification of losses in the small categories. Therefore, simply using weighting factors to solve the data imbalance problem is not effective for adversarial attacks. In this work, we attempt to use the adversarial focus loss function for training in the most common view of ViT and its variant architectures and further demonstrate its effectiveness. For comparison, we use the cross-entropy loss function as the conventional loss function for the ViT model in the baseline, and we further use the focal loss AFL function to demonstrate its advantages in dealing with the category imbalance problem.

### Quantification and statistical analysis

The deep learning models were implemented in Python 3.11 using PyTorch 2.7.1, and all computations were performed in the Python environment.Model performance was evaluated using Robust Accuracy (RA) and Attack Success Rate (ASR). All experiments were performed on the complete set of correctly classified validation images without random subsampling. APA was further evaluated through robustness analysis, ablation studies, parameter analysis, class-wise evaluation, transferability analysis, patch-level transformation analysis, and visualization analysis. Schematic illustrations were created using Microsoft PowerPoint, and all quantitative figures were generated using Python-based visualization libraries. Additional details regarding evaluation metrics, parameter settings, and experimental protocols are provided in the method details, results, figure legends, and supplementary information.
